# Optimizing and Testing an Individualized and Adaptive Physical Activity Digital Health Intervention: Protocol for a Control Optimization Trial Embedded Within a Randomized Controlled Trial

**DOI:** 10.2196/70599

**Published:** 2025-08-15

**Authors:** Meelim Kim, Shadia Mansour-Assi, Mohamed El Mistiri, Junghwan Park, Sarasij Banerjee, Owais Khan, Steven De La Torre, Michael Higgins, Job Godino, Kevin Patrick, Camille Nebeker, Sonia Jain, Predrag Klasnja, Daniel E Rivera, Eric Hekler

**Affiliations:** 1 Herbert Wertheim School of Public Health and Human Longevity Science University of California, San Diego La Jolla, CA United States; 2 The Qualcomm Institute University of California, San Diego La Jolla, CA United States; 3 The Design Lab University of California, San Diego La Jolla, CA United States; 4 Laura Rodriguez Research Institute Family Health Centers of San Diego San Diego, CA United States; 5 Control Systems Engineering Laboratory School for Engineering of Matter, Transport, Energy Arizona State University Tempe, AZ United States; 6 Ministry of Health and Welfare Korean National Government Sejong Republic of Korea; 7 Exercise and Physical Activity Resource Center University of California, San Diego La Jolla, CA United States; 8 School of Information University of Michigan–Ann Arbor Ann Arbor, MI United States

**Keywords:** physical activity, digital health, optimization trials, control optimization trial, randomized controlled trial

## Abstract

**Background:**

While effective physical activity (PA) interventions exist, interventions often work only for some individuals or only for a limited time. Thus, there is a need for digital health interventions that account for dynamic, idiosyncratic PA determinants to support each person’s PA. We hypothesize that supporting individuals with their personal PA goals requires a personalized intervention that both supports each person in forming daily habits of walking more and develops personalized knowledge, skills, and practices regarding engaging in exercise routines. We operationalized these adaptive features via a digital health intervention called YourMove that uses a control systems approach to support personalized habit formation and a self-experimentation approach to develop personalized knowledge, skills, and practices.

**Objective:**

The primary aim is to evaluate differences in minutes of moderate to vigorous PA (MVPA) per week at 12 months comparing our personalized intervention, called YourMove, with an active control that is similar but without personalization of the intervention components and mimics best-in-class digital health worksite wellness programs.

**Methods:**

The YourMove study is a 12-month randomized controlled trial that involves 386 inactive adults aged 25 to 80 years. All participants receive (1) a Fitbit Versa smartwatch and corresponding smartphone app; (2) weekly PA goal suggestions and feedback, behavior change strategies, and reminders via SMS text messaging; and (3) up to US $50 in incentives for reaching daily step goals. Participants randomized to the active control group, modeled after worksite wellness programs, receive all the elements described in addition to a static daily step goal and static point rewards. Participants randomized to the intervention group receive (1) a habit formation element with daily personalized step goals and personalized point rewards generated through a control optimization trial approach and (2) a knowledge, skill, and practice development element featuring a self-guided self-experimentation tool that helps individuals find strategies to improve MVPA. The primary outcome is objectively assessed weekly minutes of MVPA via an ActiGraph monitor.

**Results:**

Recruitment began in October 2022 and concluded in August 2024. Data collection will conclude in August 2025, with results expected by early 2026.

**Conclusions:**

We hypothesize that the intervention group will show greater improvement in MVPA than the active control group at 12 months. If the hypothesis is supported, this will provide compelling evidence to suggest that personalized and perpetually adaptive support can enhance PA more effectively than intervention elements commonly used in digital health worksite wellness programs. If the trial is successful, the results will provide justification to explore both the control optimization trial approach and self-experimentation approach for other complex, idiosyncratic, and dynamic behaviors such as weight management, smoking, or substance abuse.

**Trial Registration:**

ClinicalTrials.gov NCT05598996; https://clinicaltrials.gov/study/NCT05598996

**International Registered Report Identifier (IRRID):**

DERR1-10.2196/70599

## Introduction

### Significance of the YourMove Intervention

Strong evidence indicates that physical activity (PA) reduces the risk of a variety of cancers (eg, bladder, breast, and colon) [1-5] and cardiovascular disease [6] and improves glycemic control [7]. The 2018 Physical Activity Guidelines recommend 150 minutes per week of moderate to vigorous PA (MVPA) and emphasize taking an adequate amount of steps each day as a behavioral target [8]. However, only a third of adults meet these guidelines for PA [9-17]. As inactive adults are at high risk of developing cancers [4,5] and other chronic diseases, PA is a key behavior to target for this population [8], which suggests the need for PA interventions.

Digital-based interventions show great promise for promoting health behavior change [18], and as a result, many PA interventions are being developed via digital technology [19,20]. While effective digital health interventions (DHIs) for PA exist, many have significant limitations that result in gaps between hoped for results and actual outcomes. In particular, previous research shows that DHIs for PA have a moderate short-term effect on PA, are acceptable with good adherence when used in research studies [5,21-28], and show meaningful 12-month results with effect sizes of 0.2 relative to comparators [29,30]. However, these interventions were found to only be effective for certain populations or for a limited period [31-33]. This can be partially explained by the idiosyncratic nature of PA, with prior work indicating that individuals’ PA is influenced by different factors [34,35]. In addition, PA is dynamic in that individuals can engage in PA for a time, but then an event may occur that prompts inactivity [24,36-44]. Our prior work shows that individual (eg, stress and busyness) and contextual (eg, day of the week and the weather) factors have different relationships to PA for each person [34,35] and these predictors change over time [9-16,45]. These findings point to the need for interventions that account for dynamic, idiosyncratic PA determinants to support each person’s PA.

We refer to DHIs for PA that adapt the support provided to individuals’ unique and changing needs as personalized and perpetually adaptive interventions [24,46]. While the approach that the team has developed to create scalable, personalized, and perpetually adapting DHIs described in prior work shows promise [47,48], a randomized controlled trial (RCT) will produce valuable evidence for examining the degree to which this approach is a meaningful improvement relative to a comparator modeled after a digital health worksite wellness offering.

### Purpose of This Paper

The purpose of this paper is to describe the research protocol of the YourMove study, which tests the YourMove intervention via an RCT. This paper is organized as follows: (1) an explanation of the approach that was used in the YourMove intervention to increase MVPA by operationalizing personalized and perpetually adapting support, namely, the use of control systems methods to support habit formation with respect to walking and a self-experimentation approach to support knowledge, skill, and practice development; (2) a description of the YourMove trial methods, including study aims, participant recruitment, intervention procedures, measurement follow-up, and the statistical analysis plan; and (3) the implications of YourMove regarding future research on optimization of DHIs for PA.

## Methods

### Aims

The primary aim of the YourMove trial is to evaluate differences in minutes of MVPA per week at 12 months comparing the personalized and perpetually adapting YourMove intervention against an active control that was designed to mimic current worksite wellness offerings for fostering PA. We hypothesize significantly higher minutes of MVPA per week at 12 months compared to baseline in the YourMove intervention group relative to the control group measured via the ActiGraph GT3X+ [49-51]. The secondary aims include examining continuous daily measures (ie, steps per day) via a Fitbit Versa (Google) smartwatch [52] and daily intervention engagement. In addition, comparing baseline and 6- and 12-month follow-up measures, we will examine metabolic syndrome–related health outcomes directly related to cancer risk (ie, submaximal oxygen consumption, BMI, and body composition), psychological variables (ie, quality of life, anxiety, and depression), and the percentage of individuals that meet and maintain 150 minutes per week of MVPA.

### Study Design

The YourMove study is an RCT with a sample of 386 inactive adults aged 25 to 80 years. Participants are randomized to either the YourMove intervention or active control group. The primary outcome is change in minutes per week of MVPA at 12 months relative to baseline as measured via the ActiGraph activity monitor [49-51] using standard procedures [53,54]. Assessments of these constructs are conducted through a combination of in-person measurement at the Exercise and Physical Activity Resource Center (EPARC) at the University of California, San Diego (UCSD), and continuously via the Fitbit smartphone app and wearable PA monitor.

### Innovation of the YourMove Intervention

#### Overview

The YourMove intervention seeks to be a scalable DHI to improve MVPA by providing personalized and perpetually adapting support via (1) a control optimization trial (COT) systems engineering approach to support habit formation regarding daily steps and (2) a self-guided self-experimentation approach for developing personalized knowledge, skills, and practices to exercise regularly [48].

Prior work demonstrates the value of individuals receiving health coaching from a person. A key advantage of a human health coach is that they can listen carefully to a person to ensure that they can personalize their approach and suggestions for each individual and adapt to them over time as their needs change. These characteristics are not easily replicated within current DHIs as present-day DHIs mostly rely upon a priori decision rules and policies to guide adaptation. Within the DHI tested in this RCT, the team incorporated 2 approaches to achieve personalization and perpetual adaptation that are not common in current DHIs. One uses methods from control systems engineering to address the challenge, and the other incorporates the person themselves as the ultimate arbitrator on whether something is working for them via a self-guided self-experimentation tool. Each approach will be described, with a particular focus on how it offers personalized and perpetual adaptation that better mimics what a human coach could normally provide.

#### A Control Systems Approach for Fostering Walking Habits

This study uses a control systems approach, which the team has labeled a COT, to help people develop walking habits in a personalized and perpetually adapting way. It is based on the well-established field of control systems engineering, which is pervasive and embedded within a range of well-known technologies such as pacemakers, climate control, and robotics, yet it often goes unnoticed as a hidden technology [55,56]. Control systems engineering focuses on decision-making in systems that change over time. COT is an approach for creating personalized and perpetually adapting interventions via the development of individualized protocols for each person that has 2 key phases. The first phase is for each participant to take part in an embedded system ID [57-59] experiment (which is an N-of-1 trial design). The purpose of this initial phase is to develop personalized computational dynamic models to guide personalization. After this phase is completed, the next stage involves the use of these personalized dynamic models within a control systems engineering–based decision-making algorithm called a controller [60-62] to enable daily, perpetual adaptation.

We specifically apply our COT approach for personalization and perpetual adaptation to 2 well-recognized behavior change techniques (BCTs): goal setting and positive reinforcement. We hypothesize that this approach to personalization and perpetual adaptation can facilitate (1) improved adherence as persons will experience receiving the “right” support over time; (2) enhanced cultivation of walking habits as adjustments are made in context and over time, thus plausibly improving habit formation; and (3) increased likelihood of maintenance via the creation of resilient walking habits that, plausibly, are more likely to be maintained even after sessions of use of the intervention.

**Figure 1:**
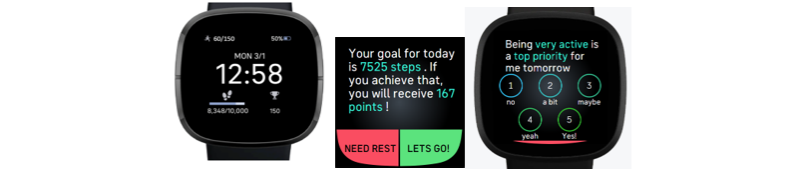
YourMove intervention Fitbit watch face, daily step goal prompts, and ecological momentary assessment.

Turning back to our health coach analogy, our COT approach for creating a personalized and perpetually adaptive DHI is an automated way to achieve many desirable attributes that, in the past, only a health coach could provide. A good health coach has several attributes: (1) a clear sense of what meaningful behavioral targets are, such as PA guidelines; (2) the ability to actively monitor a person’s behavior; (3) awareness of a person’s broader life circumstances and how those circumstances influence their behavior; (4) a repertoire of evidence-based BCTs that can be used and adapted to each person; and (5) the capacity to learn about a person and continuously adjust support based on their changing needs. Similarly to an effective health coach, our COT-based intervention has the following capabilities: (1) ambitious and achievable step goals based on participant-specific models and the performance of each participant on the most recent days that strive toward fostering steps per day that are recognized as conferring health benefits (eg, 8000 steps per day) [63-65]; (2) continuous monitoring of steps per day via a Fitbit Versa smartwatch; (3) monitoring of other facets of a person’s life (eg, weather patterns, daily stress, and busyness); (4) use of multiple BCTs (ie, goal setting, provision of feedback, positive reinforcement, self-monitoring, prompting of intended action, action planning, and education process) that adapt to a person’s changing needs; and (5) prediction of how a person will respond to support each day, with subsequent monitoring to test the accuracy of each daily prediction and results on accuracy incorporated into future predictions, thus operationalizing learning about each person. These components are delivered to intervention participants via a study-specific Fitbit watch face and ecological momentary assessment (EMA) prompts, shown in Figure 1.

Using our previously developed and vetted dynamic model of social cognitive theory (SCT) and insights from running N-of-1 system ID experiments with each person, our COT-based intervention can use continuous monitoring of steps per day and other individual factors to make predictions on each person’s response to different intervention adaptations considering a person’s current state and context. On the basis of those predictions and matching to the desired state (eg, 7500 steps per day being maintained), the system performs N-of-1 adaptations related to suggested goals, feedback offered if goals are not met, and the amount of reward given. For example, our controller can adjust (1) the suggested daily step goal and (2) the reward points offered each day when goals are met or not.

This COT approach mimics how a health coach may hypothesize about a person’s response to different BCTs. The discrepancy between a controller’s prediction and a person’s actual response is incorporated into future decisions of the controller. For example, if a person starts to be less responsive to suggested goals, the controller may increase the reward that a person will receive for meeting a goal to increase a person’s incentive to meet suggested goals. In sum, our COT approach is a robust, systematic, scalable approach for providing many key benefits of a health coach via an automated tool. The following sections provide more details on how this was operationalized.

#### Self-Experimentation to Cultivate Personalized Knowledge, Skills, and Practices

**Figure 2:**
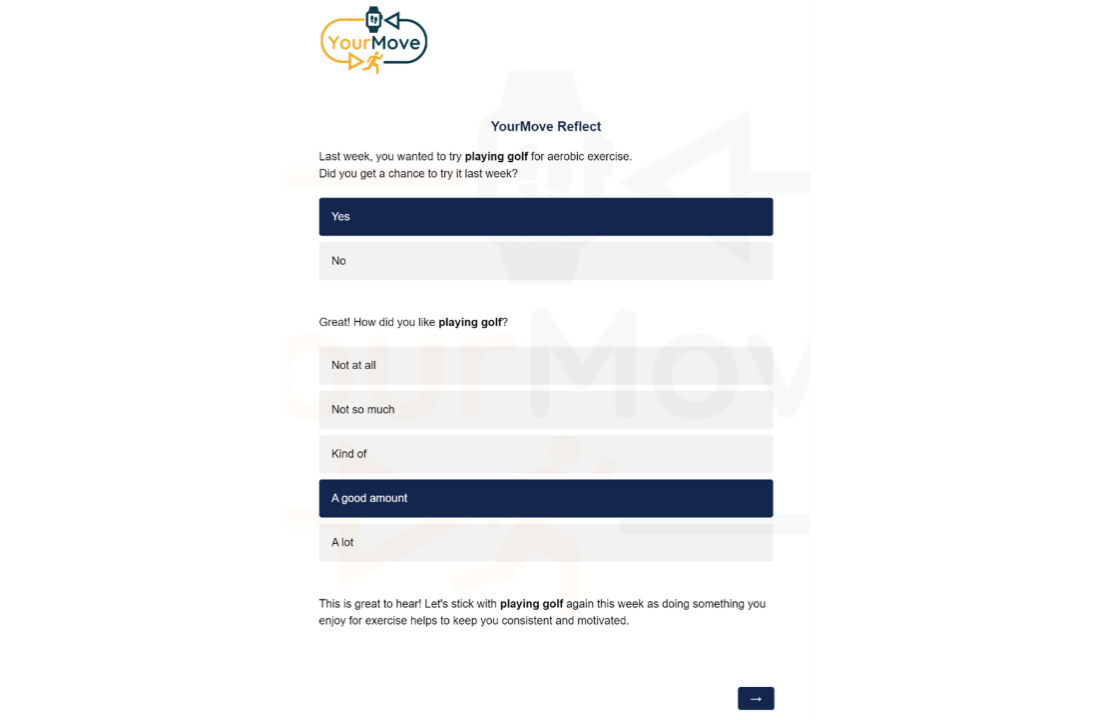
Example interface from YourMove Reflect tool.

**Figure 3:**
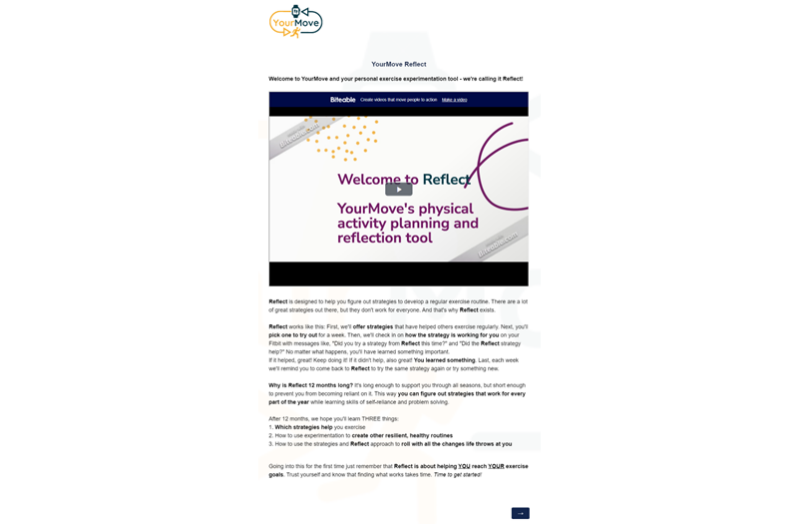
Participant introduction to YourMove’s Reflect self-guided self-experimentation tool.

In this study, we developed a self-guided self-experimentation tool titled Reflect to support participants in developing personalized knowledge, skills, and practices [48] related to exercising. To do so, the Reflect tool mimics a typical health coaching session using a logic-based survey design on Qualtrics (Qualtrics International Inc). Follow-up questions, feedback, and advice are contingent on participants’ responses, shown in Figure 2. To build knowledge and skills, participants are guided through a series of questions to identify personal MVPA goals, exercises to meet their MVPA goals (eg, walking, running, and recreational sports), strategies that work well in general for helping people engage in exercise (eg, strategies to do before, during, and after exercise), and basic instructions on how to try out the strategy for a week to see whether it works. Strategies are framed as short-term experiences, or “experiments.” Participants are asked to use personal judgment to decide which strategies to try and then use their experience while experimenting to define whether a strategy is successful. This contrasts with more common approaches such as our COT approach, which seek to have the technology define whether a person is successful. The Reflect tool is outlined for participants in a 3-minute informational video and accompanying text description to view before using the Reflect tool initially and as needed throughout the intervention, as shown in Figure 3.

**Figure 4:**
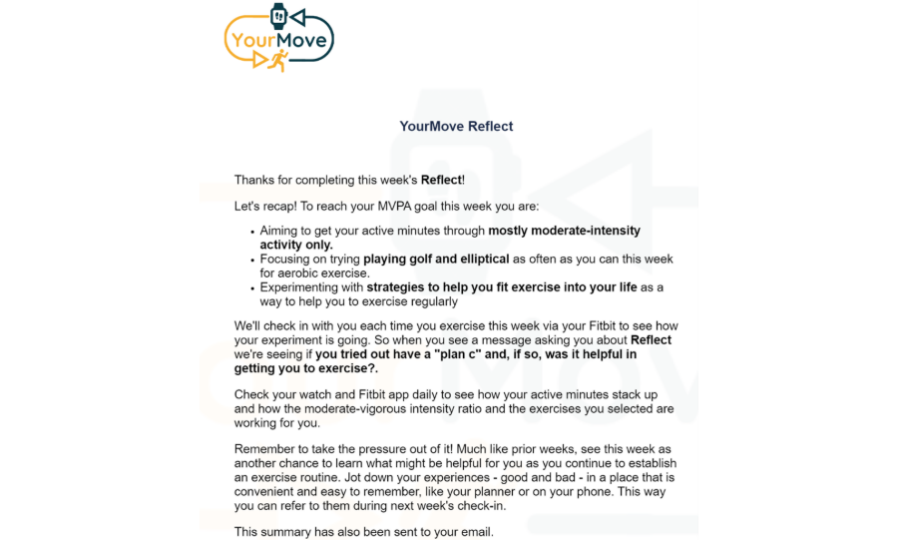
YourMove Reflect tool response summary.

**Figure 5:**
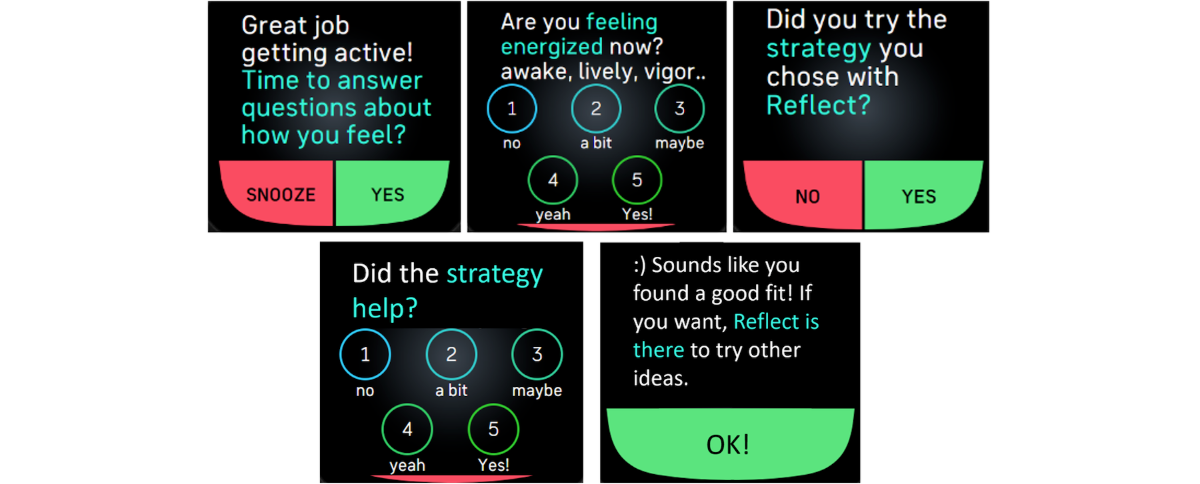
YourMove Reflect tool Fitbit watch face follow-up prompts.

Following completion of the tool, participants receive an automated summary of their developed plan for the week via email. Throughout the week, whenever a bout of exercise is detected via Fitbit, participants are prompted on their smartwatch to indicate whether they tried their Reflect strategy and, if so, whether the strategy was useful. Examples of this follow-up with participants after the use of the Reflect tool are shown in Figures 4 and 5. Depending on responses, participants are invited to return to Reflect to try out different strategies and continue with the process. For example, if the person rates the strategy as working well, this is acknowledged, and they are invited to go back and work on other strategies that might also help if they thi`nk they need more support. However, if a person rates a strategy as not helpful, they are also invited back to the Reflect tool to try something different. Through this, the system aligns with the priorities and interests of the individual, trusting them to determine whether a strategy is helpful for them. This personalized and perpetually adapting approach guides participants toward strategies that might help them and are aligned with their self-interests and evolving PA goals, much like how a health coach might do so in a session. Details about the contents of the Reflect tool can be found in Multimedia Appendices 1-7.

With these approaches, Reflect helps participants develop a personal health practice related to exercise. Contrary to habit formation, as targeted using the COT approach to improve daily steps, with Reflect we aim to cultivate an exercise practice by having participants be engaged in the process of determining what strategies work best for them and refining and personalizing strategies for themselves as needed.

### Comparator Selection

When selecting a meaningful comparator, it is critical to identify one that best serves the trial’s primary purpose [66]. To do so, we chose to compare the YourMove intervention to an active control that both could be viably deployed in real-world settings as a worksite wellness program and incorporated intervention components that, in previous literature, have been shown to be valuable for producing meaningful results in DHIs for PA. The YourMove intervention, as described, is a DHI designed to offer personalized and perpetually adapting support to both support habit formation related to walking and cultivate personalized knowledge, skills, and practices in exercising. On the basis of our already tested DHIs [35,67], the YourMove and active control groups both include education, weekly planning, PA history, motivational messaging, and implementation intentions through weekly SMS text messages. Both groups will also include the goal setting and feedback, self-monitoring, and positive reinforcement BCTs, as well as equal financial incentives (US $50) for meeting daily step goals, with the key difference being in how these 3 BCTs are provided in the 2 groups. On the basis of this, our trial provides a strong mechanistic comparison. In addition, our comparator was designed to mimic currently scaled standard-of-care DHI worksite wellness offerings. With this, the results of this trial are valuable for understanding ways for determining both whether our specific strategies for delivering personalized and perpetually adapting interventions are effective and whether they perform better than an intervention that mimics current worksite wellness offerings. Table 1 provides a summary of the key common and different features between the YourMove intervention and our active control, which was designed to control for common offerings commonly available in digital health PA interventions (eg, self-monitoring, financial incentives for meeting step goals, and receiving a Fitbit). The Active Control section provides more details on the active control condition.

**Table 1:** Summary of features in the active control and the YourMove intervention.

Intervention feature					Active control			YourMove intervention
**Digital technology tool**								
	Fitbit Versa smartwatch			✓			✓	
	Smartphone app			✓			✓	
Financial incentives—receiving up to US $50 for meeting their suggested step goals					✓			✓
**Tailored SMS text messaging support**								
	Weekly PA^a^ goal and feedback			✓			✓	
	PA behavior change strategy			✓			✓	
	Midweek PA goal feedback			✓			✓	
	Reminder to complete the Reflect tool						✓	
**Daily support**								
	**Daily step goal**			✓			✓	
		N-of-1 adapted?				✓		
	**Daily points contingent on goal being met**			✓			✓	
		N-of-1 adapted?				✓		
	Daily EMA^b^ both for self-monitoring and to measure SCT^c^ constructs that inform N-of-1 adaptation						✓	
PA reflection tool								✓
Adherence support—check-ins to support adherence to measurement					✓			✓

^a^PA: physical activity.

^b^EMA: ecological momentary assessment.

^c^SCT: social cognitive theory.

### Ethical Considerations

#### Overview

This study was approved by the Human Research Protections Program at UCSD (protocol 200733) and is registered on ClinicalTrials.gov (NCT05598996). In addition to these requirements, the study team engaged in several additional steps to ensure that the study was conducted with the highest degree of ethical practices to balance out a range of often competing requirements related to (1) ethical best practices (honoring autonomy, beneficence, and justice), (2) risks and benefits both for individuals and society at large with respect to conducting the research study, (3) data management and corresponding ethical issues inherent to commercially available digital health tools (eg, managing privacy and data sharing issues), (4) assessment of the state of the science to guide decision-making on the type of evidence production that is appropriate, and (5) system usability and fit into a person’s real-world context.

Informed consent was obtained from each participant during the baseline visit. Before this in person consent discussion, individuals were directed to a web-based screening form to review the study purpose, procedures, risks, and benefits and respond to eligibility questions. Eligible individuals were contacted by phone to verify their responses and to schedule a baseline visit. Data from Fitbit Versa devices were continuously and securely transmitted to Fitbit servers, retrieved by Fitabase, and stored on a Fitabase secure server. De-identified data were downloaded for analysis and stored on password-protected servers at the EPARC, with no personally identifiable information was linked to the device-captured data. EPARC servers were accessible only to study staff involved in the measurement of study participants and analysis of study data. Researchers using commercial devices for digital health research have a responsibility to understand and evaluate how vendors manage participant data, especially when data remain on third-party servers even after being exported to platforms like Fitabase. In this study, the decision to use Fitbit was made prior to the company’s acquisition by Google, at a time when Fitbit’s privacy policy reflected stronger consumer protections. Participants received financial incentives for both participation and measurement adherence. For the intervention phase, participants in both arms could earn up to US $50 over 12-months based on points awarded for meeting daily step goals. Intervention participants received personalized daily goals and point values generated by a control algorithm, while control participants received a fixed daily goal of 10,000 steps and static point values (150 points/day). In both groups, 1000 points equaled US $1. To encourage retention, participants received US $25 and US $50 for attending the 6- and 12-month follow-up measurement visits, respectively. Thus, participants could earn between US $75 (measurement incentives only) and US $125 (maximum incentives for both measurements and step goals). Participants who completed at least one month of the study were allowed to keep the Fitbit Versa device (valued at US $230) A full review of the ethical and design work that the team conducted to prepare this study is beyond the scope of this protocol paper but is an important and complementary set of activities that was a central part of this study. Key activities that the team conducted included (1) active use of the Digital Health Checklist [68] as a general guide for thinking through and making initial decisions about technology selection, design decisions for the technology, and decisions about other ways of balancing the often competing demands outlined previously; (2) conducting a series of human-centered design workshops that invited prospective participants to engage with and provide the team with feedback on possible design decisions for the technology (eg, selection of the Fitbit vs other devices and design options that balance the desire for honoring the autonomy of participants while minimizing burden); and (3) applying for and receiving a bioethics administrative supplement specifically focused on developing guidance specific to strategies for improving consent communications about the complexities of the control algorithm to participants. The consent research aimed to enable prospective participants to make truly informed decisions about the use of digital technologies in their own lives [69]. The results of the first 2 activities are summarized in the following section, and the results of the third set of activities are reported elsewhere [69-72].

#### Summary of the Results of Engaging With the Digital Health Checklist

Overall, our activities resulted in the selection of the Fitbit based in part on the ethical and data management practices that are used at Fitabase, which was the digital health technology company that ultimately operationalized many of the key elements of our overall study protocol. In addition, our team devised a robust and comprehensive set of activities that were all designed to ensure that participants engaged with sets of questions to assess potential risks of participating (which largely related to potential risks that arise if a person has health challenges that would result in increasing the likelihood of adverse events due to exercising) and also helped participants make informed decisions, including the use of a motivational interviewing–style approach to informed consent that involves asking participants to develop their own personal list of the pros and cons of both participating and not participating in the study before making a final informed consent decision. In addition, our entire algorithm development process was explicitly established to be able to align with and learn each person’s priorities, interests, and goals and, from there, align with a person’s context and needs. This was done via the mixed use of both the COT approach for creating a personalized (via system ID) and perpetually adapting (via the control algorithm) approach that is all enacted in a purely idiographic (ie, N-of-1) way. This approach reduces the risk of a systemic bias of an algorithm developed nomothetically (eg, an algorithm that was trained on persons with largely light skin creating a systematic bias that reduces confidence in the results when used with persons with darker skin) as there is no requirement for all participants to have the same computational algorithm to guide personalization or the same enactment of the control algorithm to support personalization. Moreover, we included our self-experimentation Reflect tool with the explicit goal of inviting persons to cultivate their knowledge, skills, and practices for exercising, with the ultimate judge on whether a strategy is a good one being the person themselves. Through examining the key questions and challenges of the Digital Health Checklist, the team concluded that we had produced a digital health tool that could be implemented in a way that, most critically, honors individual autonomy; leans toward beneficence in the form of aligning the success of the intervention strategies directly with the goals of the person, accounting for their personal assets and constraints; and honors justice in the form of advancing an algorithmic and self-experimentation approach that, at its core, can align with each person’s unique needs while also being automated and, thus, scalable, thus providing a pathway for advancing justice in the implementation of digital health tools.

### Eligibility Criteria

The targeted population were inactive adults interested in increasing their weekly PA. Inclusion criteria were (1) age of 25 to 80 years; (2) intention of being available for a 12-month research study; (3) willingness and ability to attend 3 measurement visits at UCSD over the 12-month RCT; (4) PA deficiency (<150 MVPA minutes per week and <75 vigorous PA minutes per week based on the International Physical Activity Questionnaire) [73]; (5) passing the conditional logic of the Physical Activity Readiness Questionnaire–Revised (PAR-Q) [74], which screens for PA readiness, OR being approved by their physician if they did not pass the PAR-Q; (6) BMI between 18 and 40 kg/m^2^; (7) willingness and ability to use a smartphone and SMS text messaging; (8) willingness and ability to use the Fitbit Versa smartwatch and app; (9) willingness and ability to walk and engage in moderate-intensity activities; and (10) ability to speak English. Exclusion criteria were (1) a mechanical medical implant, (2) enrollment or plans to enroll in a PA program during the study period, and (3) psychiatric or medical conditions that prohibited compliance with the study protocol.

### Recruitment, Enrollment, and Retention

A diverse sample of prospective participants were recruited in the San Diego region via digital advertisements, targeted social media advertisements, posting of print and digital flyers at UCSD and community centers, emails sent by community public health partners, and county-wide events via tabling. Interested individuals were directed to complete a web-based screening form through the study’s secure REDCap (Research Electronic Data Capture; Vanderbilt University) database hosted at UCSD [75] that provided further details about the study and criteria for participating. Individuals were then asked eligibility questions, including their age, BMI, and current PA behavior via the International Physical Activity Questionnaire [73], and study staff followed up accordingly.

**Figure 6:**
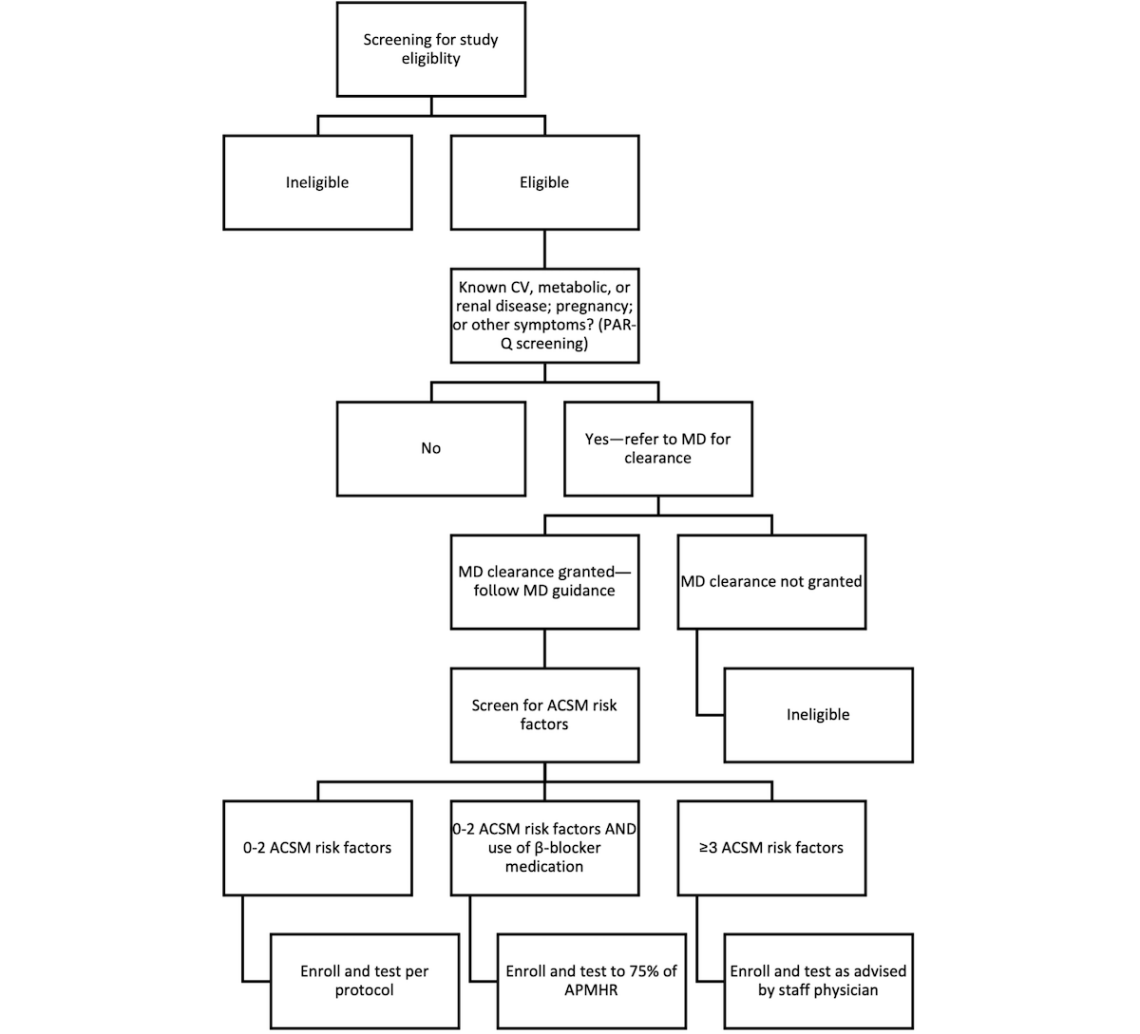
Pre-enrollment screening and exercise testing procedures for YourMove. ACSM: American College of Sports Medicine; APMHR: age-predicted maximal heart rate; CV: cardiovascular, MD: medical doctor; PAR-Q: Physical Activity Readiness Questionnaire.

Prospective participants who did not meet the eligibility requirements received an email notifying them and thanking them for their interest in the study. Prospective participants who met the eligibility requirements received a call from a trained research staff member notifying them that they may be eligible to participate, informing them about the study details, explaining that they may be randomized to an active control group, and confirming the information provided in the web-based screening form. Because participants engaged in a submaximal treadmill test, those eligible were also prescreened for exercise testing using the PAR-Q [74]. Those who did not pass the conditional logic of the PAR-Q were required to obtain approval from a physician before participation. In addition, prospective participants who reported ≥3 American College of Sports Medicine risk factors [76] were referred to the study physician to determine how to proceed with exercise testing. The study physician determined whether the participant could (1) take part in exercise testing as normal, (2) take part while being monitored by an electrocardiogram, or (3) take part with clearance from the participant’s physician. Once prescreening procedures, shown in Figure 6, were completed and the prospective participant was deemed eligible, they were invited to schedule a baseline measurement appointment at the EPARC at UCSD. This entire prescreening process reflected our commitment to truly informing our prospective participants of the opportunities and potential risks, with risk assessment taking place on their behalf to ensure that they had all the information they needed to decide. With this information, prospective participants were then provided with the final written informed consent document to review it and ask questions about the study; if they agreed, they signed the consent form to conclude the enrollment process. Following written consent, the baseline measurements took place.

After eligibility, consent, and BMI were confirmed and baseline measurements were completed, participants were randomized to either the YourMove intervention or the active control at a ratio of 1:1 stratified by sex. The biostatistics team generated an electronic randomization list using the latest version of the statistical software platform R (currently version 4.1.2; R Foundation for Statistical Computing [77]). The pseudorandom seed was saved so that the code could be validated and the sequence regenerated if necessary.

To meet enrollment targets and increase retention, each participant was provided with a Fitbit Versa smartwatch and gift card incentives throughout the study. At the baseline measurement visit, after enrollment and randomization, participants received a Fitbit Versa. The device is theirs to keep upon completing at least 1 month of participation in the study. To maximize the likelihood that participants will continue with follow-up measurement visits and stay in the study, US $25 and US $50 gift cards are provided at the completion of the 6- and 12-month measurement visits, respectively, for a total of US $75. Participants in both the YourMove intervention and active control groups also receive up to US $50 over the 12-month study contingent upon meeting their assigned step goals and based on the number of points assigned to them each day. For the YourMove intervention, goals and points are assigned based on, first, the system ID experiment (to generate a personalized computational model) and then on the control algorithm to support perpetual adaptation (which, overall, we refer to as the “COT approach,” a term that we will use when referencing the overall approach regardless of phase). Daily step goals for each participant are estimated to be challenging for the person but also possible based on their step data history and predictions from the COT approach. In addition, daily points are assigned by the COT approach, with the phase 2 control algorithm set to lean toward providing the fewest number of points needed to facilitate a person meeting their step goals on any given day. For the active control, daily step goals are 10,000 steps per day. Points remain static at 150 points per day. For both groups, 1000 points equal US $1, and the maximum amount that each participant can earn over the course of the 12-month study is US $50. The minimum monetary compensation that a participant can receive for completing the study (both in the intervention and control conditions) is US $75 by completing only the measurement visits, and the maximum monetary compensation is US $125 by achieving the maximum of US $50 for reaching the daily step goals and US $75 for completing each measurement visit.

### Measurement Protocol

#### Overview

Outcomes are measured at baseline and the 6- and 12-month follow-up. The primary outcome measure, minutes per week of MVPA, is assessed via the ActiGraph GT3x+ [49-51] using standardized procedures [50,53]. Participants are instructed to wear the device continuously during their waking hours for 7 days except while bathing or swimming. Minute-by-minute estimates of activity are categorized into intensity-weighted summaries (ie, sedentary, light, moderate, and vigorous) of PA using previously validated calibration thresholds as well as cutoff points used in national surveillance systems [49,50]. Steps per day are also determined using a proprietary algorithm from ActiGraph that has been independently validated [51].

Secondary outcome measures include anthropometric and physiological measures, behavioral measures, psychological measures, related lifestyle factors, and overall participant experience and satisfaction. Anthropometric and physiological outcomes are measured using the following standardized procedures and conducted by trained staff: (1) body weight is measured to the nearest 0.1 kg using a calibrated digital scale; (2) height is measured to the nearest 0.1 cm using a stadiometer; (3) BMI is calculated from the height and weight measurements as kg/m^2^; (4) waist and hip circumference is measured to the nearest 0.1 cm using stretch-resistant measuring tape; (5) body composition is measured through bioelectrical impedance analysis using a Tanita DC-430U dual-frequency total body composition analyzer; (6) grip strength is measured to the nearest 1 kg using a grip strength dynamometer (JAMAR; model 081669928); (7) flexibility is measured using a sit-and-reach box; (8) gait speed is measured using a 25-foot walk test; (9) the Short Physical Performance Battery and Timed Up and Go test will be conducted in participants aged ≥50 years to assess lower extremity strength, transitional movements, balance, fall risk, and mobility; and (10) the submaximal graded exercise test is conducted on a treadmill using a walking protocol.

Behavioral measures include the Global Physical Activity Questionnaire; Physical Activity Neighborhood Environment Scale; Sedentary Behavior Questionnaire; Social Support for Exercise Survey; and other surveys assessing sleep, perceptions and intentions related to exercise, and PA change strategies used in previous behavioral health research. Psychological measures include the Rosenberg Self-Esteem Scale, Center for Epidemiological Studies Depression Scale, Spielberger State-Trait Anxiety Inventory, and Quality of Well-Being Scale. Other related lifestyle factors are measured using the Social Determinants of Health Toolkit, RAND Homelessness Survey, and Customary Drinking and Drug Use Record. Participant experience and satisfaction is measured using the Twente Engagement With eHealth Technologies Scale and the User Burden Scale.

**Figure 7:**
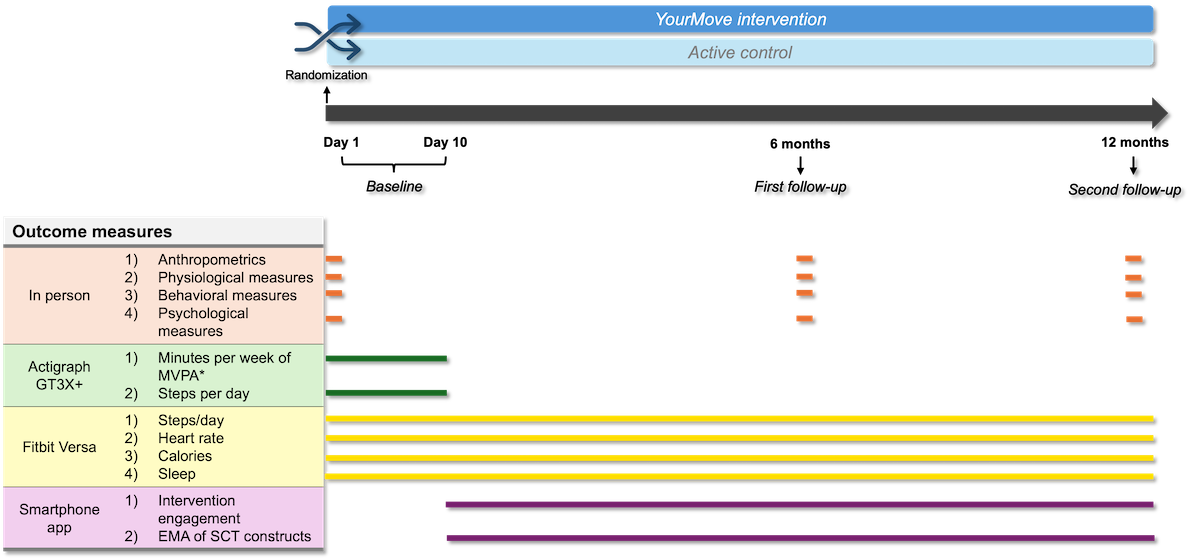
A timeline for outcome measurement. *Primary outcome measure; EMA: ecological momentary assessment; MVPA: moderate to vigorous physical activity; SCT: social cognitive theory.

Continuous measures include PA, intervention engagement, and SCT constructs and key process variables. PA is measured as steps per day and minutes of MVPA through the Fitbit Versa smartwatch. Quantitative markers of engagement for all participants include use of the Fitbit smartwatch and smartphone app and progress toward daily step goals and accompanying accrual of points. Additional markers of engagement for participants receiving the YourMove intervention include adherence in responding to daily EMAs. Participant responses to daily EMAs enable measurement of SCT constructs for our dynamic SCT model, described in the YourMove Intervention section, and key process variables. Key SCT constructs and process variables include (1) behavioral outcomes (ie, sleep, mood, physical fitness, and physical appearance), (2) busyness, (3) stress, (4) typicalness of the day, (5) internalized cue to action, (6) commitment to goal, (7) self-efficacy for meeting the daily goal, and (8) outcome expectancies (ie, sleep, physical appearance, mood, concentration, and physical fitness). The timeline for outcome measurement are shown in Figure 7.

#### Fitbit Versa Smartwatch and Smartphone App

All participants receive a consumer-level Fitbit Versa smartwatch and corresponding smartphone app. The Fitbit Versa is a wrist-worn device that objectively measures PA through its triaxial accelerometer, optical heart rate monitor, and altimeter. Upon syncing the device, participants can view PA measurements, including steps and MVPA, on the Fitbit smartphone app. Data collected from the Fitbit smartwatch are passively and securely streamed to the Fitbit website. They are then retrieved using Fitabase [78], a web-based application developed by Small Step Labs LLC. This allows for the simultaneous collection of high-resolution Fitbit data from large numbers of participants and integration with the COT approach controller and SMS text messaging system. Fitbit was chosen as the digital technology tool as it was deemed the most viable technology platform that balanced the various constraints of the study with respect to privacy, access and usability, data management, and risks and benefits. The digital health checklist mentioned previously was used to guide decision-making specific to our wearable device selection.

#### Financial Incentives for Meeting Daily Step Goals

On the basis of a review of worksite wellness programs and the literature [79-81], we found that it is plausible to assume that worksite wellness offerings will provide both wearable devices to employees and financial incentives for meeting health targets. On the basis of a review of options (coupled also with our design work described previously), we determined that US $50 in financial incentives for meeting step goals was a fiscally viable target that could be scaled and implemented at scale within worksite wellness offerings. On the basis of this, participants in both the YourMove intervention and active control are eligible to receive up to US $50 for meeting their suggested step goals. In both groups, 1000 points translate to US $1, but the approach for establishing the daily suggested step goal and number of available points varies across the 2 groups, described in detail in the following sections.

#### Tailored Text Messaging Support for Exercising

All participants receive a weekly exercise goal and feedback, behavior change strategies, and reminders to wear the Fitbit Versa smartwatch via the Fitabase SMS text messaging system. In total, 3 to 4 SMS text messages are delivered each week and follow a consistent weekly schedule, shown in Multimedia Appendix 8. Each week, participants are encouraged to achieve 150 minutes of MVPA. While this static goal is not adjusted dynamically as with the controller-generated daily step goals, participants receive personalized, tailored feedback on progress toward reaching the goal based on data collected from the Fitbit Versa smartwatch. In addition, in instances in which a participant exceeds the goal, tailored feedback encourages achieving 300 minutes of MVPA, which is associated with additional health benefits [63-65]. The behavior change strategies focus on physiological characteristics, behavioral drive, motivation, opportunity, and resiliency conditioning. Finally, if no data have been collected from the smartwatch, participants are reminded to wear their watch in lieu of weekly midweek and end-of-week feedback.

### YourMove Intervention

#### Overview

For the week following randomization, participants are instructed to engage in their normal level of PA to establish a baseline measured by wearing the ActiGraph activity monitor and Fitbit smartwatch. During this time, participants do not receive any intervention components. Afterward, participants assigned to the YourMove intervention group receive (1) the Fitbit Versa and corresponding smartphone app, (2) daily personalized step goals with corresponding point rewards generated using our COT approach, (3) the weekly PA goal (150 minutes of MVPA per week) with accompanying motivational and informative content via SMS text messaging, and (4) the Reflect tool delivered via Qualtrics. Each of these components is discussed in detail in the subsequent sections.

#### The COT Approach for Setting Step Goals and Point Rewards

**Figure 8:**
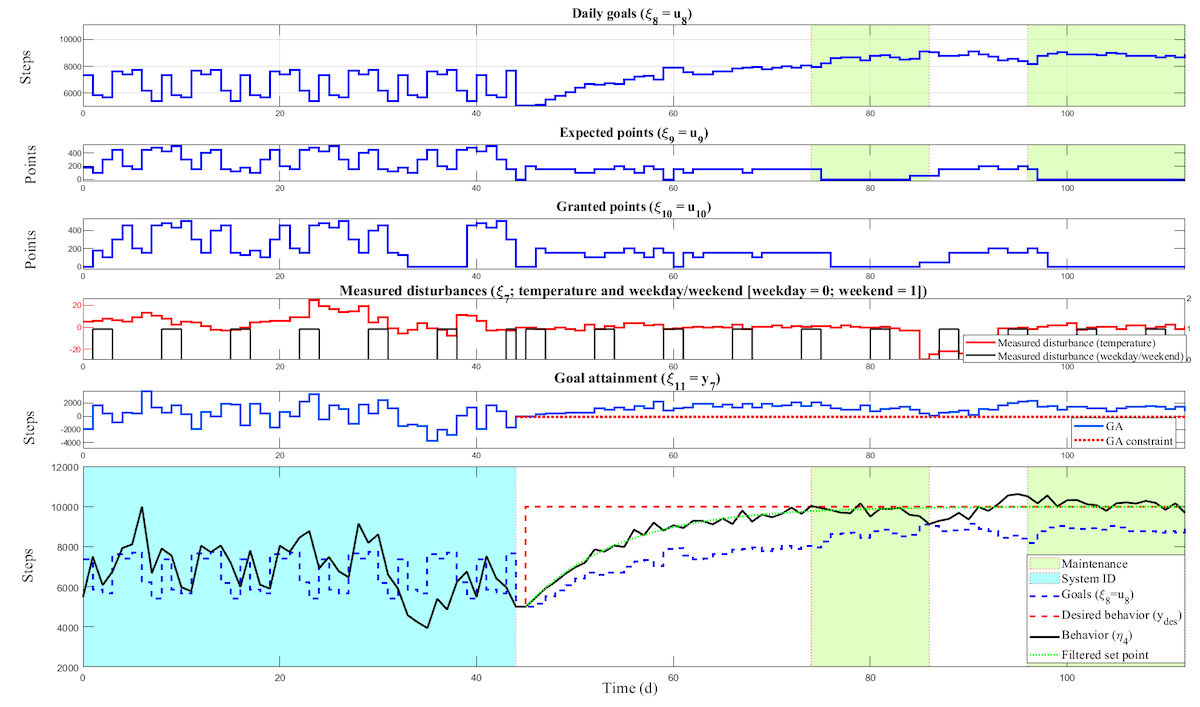
Control optimization trial simulation illustrating the system ID (cyan) and control phases for a representative participant. White regions denote the controller initiation state, whereas green regions denote the maintenance state. GA: goal attainment.

Our COT approach for creating a personalized and perpetually adapting DHI includes three phases: (1) baseline (to obtain an initial estimate of the median steps per day that the person engages in, which is the starting reference that is used in the next phase), (2) system ID (to enable the development of a computational model for each person that could be used in the next phase to provide personalized support), and (3) controller (which can use insights gleaned from the previous 2 phases to support perpetually adapting support, including the possibility of a person moving between states of behavioral initiation and maintenance of PA). On the basis of this, each phase sets step goals and point rewards differently (shown in Figure 8).

##### Phase 1: Baseline

The purpose of this phase is to obtain an estimate of a person’s median steps per day before receiving any active intervention save for the Fitbit Versa itself. To do this, participants are asked to wear the Fitbit smartwatch for 10 days but otherwise do not receive any other type of support. Participants are also asked to engage in their normal PA activities to the best of their abilities. Neither suggested daily goals nor points are provided to participants during this phase.

##### Phase 2: System ID

The purpose of phase 2 is to create a personalized dynamic model that is meant to represent each person related to engaging in steps per day. Thus, phase 2 of our overall COT approach is how we operationalize our approach to personalization.

In phase 2, an “open-loop” system ID experiment is conducted. This approach is “open loop” because the pseudorandom input signals used to define the suggested step goals and points that a person receives each day do not change based on whether a person meets their suggested step goals (it becomes a “closed-loop” response when a person’s capacity to meet suggested step goals is considered, which occurs in phase 3).

In the open-loop system ID experiment phase, each person receives their own suggested step goals and points each day, and each day, the suggestions are experimentally varied. For example, if a person’s median steps per day are 4000 (determined from phase 1 initially), then a person, over a 22-day cycle, some days will receive suggested step goals that are experimentally varied and could fit within the range of their median steps (eg, some days they are suggested to walk 4000 steps) and some days will receive more challenging step goal suggestions (eg, striving for double their baseline median, targeting 8000 steps per day). In addition to step goals, each person receives experimentally varied expected points that that they could receive if they meet their suggested step goal for that day, with the range of expected points being between 0 and 500 on any given day. Thus, for example, one day a person may not receive any points for meeting a goal, whereas on other days, they may receive 500 points for meeting a suggested step goal. In total, 1000 points equal US $1, and the accumulation of 5000 points enables a person to cash in their points for a gift card (up to US $50 in total over the 12-month intervention period). The dollar value was selected in part as it is an amount that has been used in worksite wellness programs and other offerings, thus establishing a plausible incentive structure that could be scaled and that was also used in the control group.

Step goals are minimally personalized in that they are all calculated in reference to a person’s median steps per day. Thus, for example, in the first cycle of the system ID experiment, the 10 days of the baseline phase are used to calculate the median steps per day as the reference for defining the lower range of a person’s suggested steps per day goal. Points are not personalized in this phase (eg, a person receives suggested points each day ranging from 0 to 500 points).

Pseudorandom input signals are used to dynamically and experimentally vary suggested step goals and points. Within a system ID experiment, a key concept is the notion of a cycle. A cycle is used to define the length of time needed coupled with other constraints (discussed in the following section related to input signal design) to ensure sufficient excitation of variance at a sufficient length of time to generate a computational model of the dynamic system for each individual. For this, key considerations include designing an input signal within a given cycle to have confidence that enough variations (some days with high goals, some with low suggested goals, and some with intermediate goals and, similarly, some days with a high number of points, an intermediate number of points, or no points) exist to create an estimate of the influence of steps goals and points for each person over time and across variations in context. In addition, a cycle needs to be long enough to ensure that the 2 intervention input signals are orthogonal to one another and not aligned with any known temporal patterns (eg, they do not align with a 7-day weekly cycle, which would make it impossible to disambiguate the influence of the intervention option relative to the impact of day of the week).

Within system ID experiments, replication occurs in the form of repeating the cycle multiple times. With this, the dynamic system model for each person can be produced, with cycles used to estimate the dynamic system model and then validate the predictions made for each, in line with previous protocols and strategies used and described by the study team [34,35,47]. As described in the following section and in prior work, the team engages in a sort of power calculation but with the focus not on estimating the number of participants needed but, instead, on the length of a cycle and total number of cycles that would be needed to produce a reliable and valid dynamic model for understanding and predicting each person’s walking behavior.

Once data are collected for the last day of the system ID phase, auto-regressive model with exogenous input [57-59] data analysis takes place to produce the dynamic system model for each person as our concrete approach for personalizing the YourMove intervention to each participant. Auto-regressive models with exogenous input are dynamic extensions of basic regression models. This modeling approach paired with discrete simultaneous perturbation stochastic approximation [82] provides the means to identify key variables that are predictive of each person’s steps per day over time [83]. Discrete simultaneous perturbation stochastic approximation, as described in the team’s prior work [84], is an analytic technique that can be used to overcome the need for exhaustive computational searches of all possible dynamic system models for each person. This is important because exhaustive searches (which was the team’s initial approach to this [34,35]), when scaled, result in a level of computational load that would be impractical. With this final step, the team has produced dynamic models in a way that can be fully automated and fit into real-world constraints (eg, the team was able to run all required analyses and computations for all participants in the trial overnight to ensure that every person received their own personalized suggestions every day). Through this, we have not only produced a scientifically plausible approach but also created the technology infrastructure and sets of methods that would be required for the full scalability of our approach. More details on this approach within YourMove have been published elsewhere [83,85,86].

##### Technical Details Regarding the Input Signal Design

At a high level, the basic idea of our input signal design is to experimentally vary suggested step goals and points for each person over time. The general logic is that, if one wants to understand the impact of changing something, they need to vary it, particularly across variations in context.

Turning to the technical design of this approach, in this open-loop system ID experiment, pseudorandom multi-sine input signals are designed for each of the independent intervention components (eg, goal setting and expected points). Following the guidelines presented in previous literature on systems archetypes [87], the effective frequency range can be specified by selecting the design parameters based on the estimated range of the dominant time constant of the system, 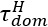 and 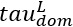, and other user-defined parameters to dictate the covered high- and low-frequency content (α_s_ and β_s_, respectively), as seen in equation 1:


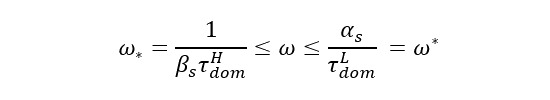


In this equation, ω_*_ and ω^*^ represent the lower and upper bounds of the effective frequency range. Input signal design is an iterative process that requires some knowledge of the system dynamics of interest. In this work, a priori knowledge from analyzing the experimental data from previous studies, particularly the JustWalk intervention [35], is leveraged. In YourMove, the selected guideline parameters for the estimated dominant time constant have been set to 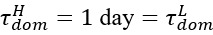. Moreover, to cover a wide range of low- and high-frequency content, the values for the user-defined coefficients have been chosen as β_s_=3.5 and α_s_=1.

**Figure 9:**
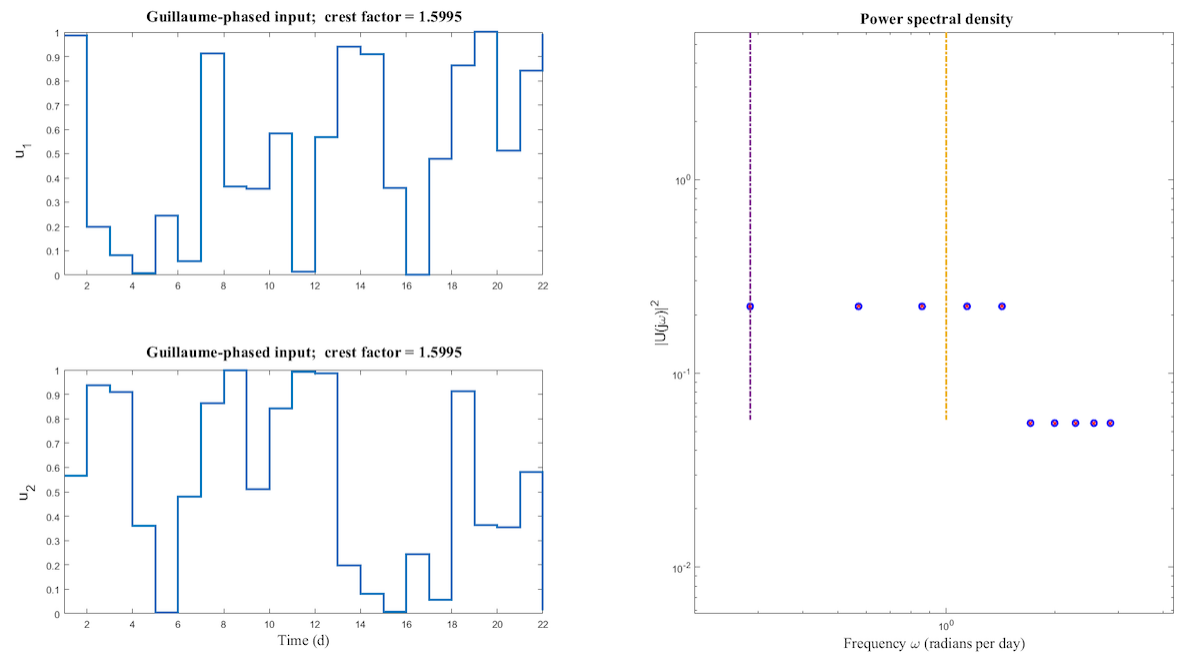
Single cycle of shifted multi-sine signals used in the system ID phase—(A) scaled time domain and (B) power spectral density.

YourMove’s input signal is designed using a shifted approach, which implies that it is generated for only 1 input and then shifted relative to the other inputs to increase statistical independence. Shifting of the input signal introduces differences in phase that promote orthogonality. The advantage of this approach is that it results in shorter cycles than the zippered approach, which was applied in the JustWalk study [35], for the same number of excited harmonics. As can be observed in Figure 9A, the overall duration of the designed input signals was selected as 22 days to allow for the execution of more cycles designated for the system ID experiment within the duration of the study.

This cycle length was also selected to allow for 5 excited harmonics in the designed input signals, as shown in Figure 9B. Because the signals for the 2 input channels are shifted copies of one another, they share the same Fourier coefficient values for all harmonics. Consequently, both signals have equally excited harmonics at the same frequencies, as can be observed in Figure 9B from the overlapping sinusoidal harmonics.

##### Phase 3: Controller

The final phase of our COT approach is when we “close the loop” and explicitly seek to offer personalized and perpetually adaptive support to each person. This is all done via the use of a controller, as used within control systems engineering. The key design focus of the controller is to monitor and be capable of supporting individuals in flowing between what the team labeled as “initiation” and “maintenance” states. By an initiation state, we refer, conceptually, to states in which a person’s walking habit is either in development or not observed as stable. By a maintenance state, we refer, conceptually, to a state in which a person appears to be engaging in a regular walking habit in that they engage in meeting targeted levels of suggested steps per day goals appropriate for their age group with minimal to no support provided by the YourMove intervention (ie, minimal suggestions related to goals and steps, including some days when neither is provided to a person).

Critically, the controller treated these as states, not stages. Through this, the controller did not make any assumptions about the possible continuation of engaging in the maintenance state in perpetuity once an initial detection of a person meeting the maintenance state was observed. This decision was made based on the well-observed phenomenon of persons lapsing (having brief moments when they no longer meet their targeted step goals but relatively quickly return to the maintenance state), relapsing (moments when a person no longer meets targeted step goals and this lack of achievement then continues), and prolapsing (moments when a person no longer meets a targeted step goal but then translates that moment into a sort of teachable moment that results in a strengthened walking habit). In addition, the team wanted to ensure that the controller could be responsive to known variations that occur in a person’s life, such as becoming sick, going on vacation, or other things (which a person can report each day after a step goal is suggested; see Figure 1 and the prompt to “take rest,” which then asks a follow-up question on reasons for rest, including illness and vacation). While this approach was used throughout, it was particularly valuable and important during the controller phase to enable personalization and perpetual adaptation.

The general logic of the control algorithm is that it uses a person’s previous data on steps per day with a particular emphasis on the previous 7 days coupled then with the use of the personalized dynamic model made for each person to run a series of simulations. These simulations vary plausible suggested step goals and points that could be provided to the person for the following day and into the future. Simulations also include seeing plausible intended and unintended consequences of different decisions that the controller could make on a person’s activity weeks and months into the future. With all these simulations run, the controller then selects an intervention (ie, a suggested daily step goal and corresponding points) that it estimates as the most likely to help a person develop a walking habit while not overwhelming them or inspiring them to give up on the use of the overall YourMove intervention. The controller does this every night and, based on this, decides every day. Thus, each day becomes a sort of mini experiment that the controller engages in. Over time, this information on the predictive capacity and ability of the controller to engage in decisions that result in helping a person develop a walking habit without overwhelming them (eg, having them stop using YourMove) is incorporated into the controller’s simulations. With this, the controller is capable of enacting a highly personalized and perpetually adaptive approach for supporting individuals in developing walking habits.

Turning now more toward the technical details that undergird the controller, first, it is critical to note that the decision-making control algorithm operates differently in each state. For behavioral initiation, the decision-making algorithm strives for appropriate daily step goals using the general approach described previously. When it comes to the behavioral maintenance state, the decision-making algorithm switches from not only seeking to optimize for steps to also minimizing the use of the financial reward component while steps are maintained. This process requires an additional logical condition that is related to consistently achieving desired levels of healthy behavior or, put more plainly, our approach for operationalizing when a person is or is not in the maintenance state. We defined a person as being in the maintenance state when they consistently reach ≥9500 steps per day for 6 out of the previous 7 days. When this condition is met, the point intervention component is reduced or deactivated by adjusting the decision-making algorithm’s tuning parameters. On the other hand, the positive reinforcement intervention component can be reactivated when the logical condition is no longer met, enabling responsiveness to lapse [88,89]. The decision-making algorithm bases its reduction of interactions on behavioral theory, specifically a thinning reinforcement schedule of points and other strategies (eg, minimize suggested goals each day to internalize targets) to help facilitate habit formation [90-92].

When there is a discrepancy between the decision-making algorithm’s prediction and a person’s actual response, the decision-making algorithm will systematically use approaches that the person appears more responsive to. These approaches are applied through a robust three-degree-of-freedom Kalman filter-based hybrid model predictive control (3DoF-KF HMPC) [85,86,88,89], biasing the decisions toward what a person has been responsive to most recently (instead of what worked earlier in the intervention). The optimization objective function for the controller is expressed in equation 2:




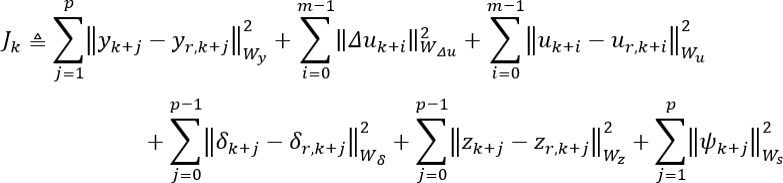




In this equation, 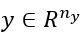 denotes the outputs, and 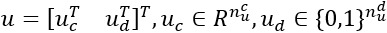 represent controller decisions with both continuous and discrete elements. 
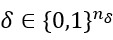
 and 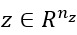 are binary and discrete auxiliary variables that convert discrete and logical decisions into their equivalent linear inequality constraints represented in equation 3:

*E*_5_ ≥ *E*_2_δ*_k_* – *E*_4_*y_k_* – *E*_1_*u_k_* + *E*_3_*z_k_* + *E_d_d_k_* – *E*_6_*u_k_*_– 1_ – *E*_7_*y_k_*_– 1_


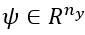
 in equation 2 represents the slack variables implemented to soften output constraints. The desired target for each variable is described by *_r_. Matrices *W_y_*, *W*_Δ_*_u_*, and *W_s_* are the penalty weights on the output error, move size, and slack, respectively, whereas *W_u_*, *W*_δ_, and *W_z_* are the penalty weights on manipulated variables, auxiliary binary variables, and auxiliary discrete variable targets, respectively. The move horizon and the prediction horizon are denoted by *m* and *p*, respectively.

On each day of the closed-loop intervention, denoted by *k*, mixed-integer quadratic programming is used to make personalized intervention decisions by solving the optimization problem in equation 2 subject to the logical and categorical constraints presented in equation 3 and the following input and output constraints:

*y*_min_ – Ψ*_k_*_+_ *_j_* ≤ *y_k_*_+_ *_j_* ≤ *y*_max_ + Ψ*_k_*_+_ *_j_*, j=1,...p

*u*_min_ ≤ *u_k_*_+_ *_i_* ≤ *u*_max_, i=0,1,...m-1

Δ*u*_min_ ≤ Δ*u_k_*_+_ *_i_* ≤ Δ*u*_max_, i=0,1,...m-1

Ψ*_k_*_+_ *_J_* ≥ 0, j=1,...p

**Figure 10:**
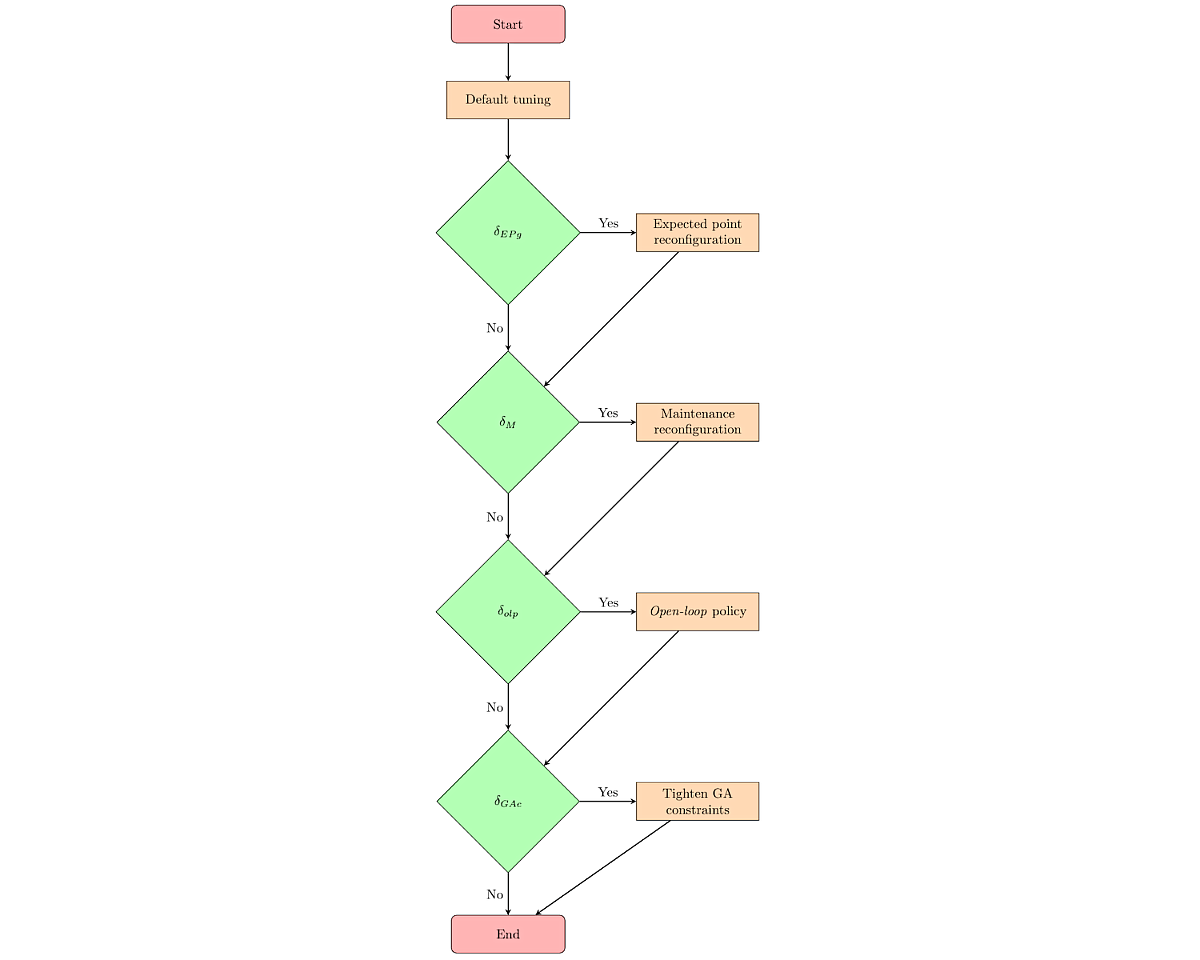
Summary of the logic used in controller reconfiguration and tuning. GA: goal attainment.

Variations in the control strategies are implemented at different stages of the closed-loop intervention as part of the decision-making algorithm. This is achieved by reconfiguring the controller and adjusting tuning parameters based on the participant’s performance in the previous 7 days of the intervention. Figure 10 summarizes the “digital PA coach tuning” algorithm used in this study. A more detailed description of the algorithm is presented in previous literature [85,93].

In addition to maintenance reconfiguration, the devised decision-making algorithm tightens the constraints based on how many days that person failed to meet the daily goals to ensure the delivery of *ambitious yet achievable* goals. For example, if the participant misses the goals for 3 to 4 days, the decision-making algorithm softly tightens the constraint for goal attainment. If the person misses the goals for ≥5 days, the decision-making algorithm constrains the daily goal to a maximum of 2000 steps per day above the average of the previous 7 days. In sum, the decision-making algorithm in our study is a robust, systematic, scalable approach for providing many key benefits of a lifestyle coach via an automated tool.

#### PA Planning and Reflection Tool

Participants are prompted to complete a self-guided self-experimentation tool titled Reflect via Qualtrics [94] weekly for the first 8 weeks and monthly thereafter. In addition, Reflect is available for participants to complete more often if preferred. At the start of the survey, participants are asked to reflect on their progress toward their weekly exercise goal delivered via SMS text message. Next, participants are provided with a list of various types of exercises that can be done to achieve MVPA (eg, biking and circuit training). On the basis of their selections, participants are then prompted to create a plan to reach their weekly goal. Each time the tool is completed, participants have an option to keep their exercise choices the same as their previous selections or make changes depending on their previous experiences. Participants then choose which exercise strategy they would like to experiment with over the following weeks. As explained to participants, the strategies are designed to help achieve the previously developed exercise plan and, ultimately, reach the weekly exercise goal. Each time a participant completes the tool, they are asked to reflect on their experiences experimenting with the strategy and, contingent on their reflections, prompted to either continue experimenting with the strategy or try another. Following the completion of Reflect, participants are emailed a summary of their responses to encourage adherence to their exercise plan and strategy experimentation. In addition, EMA messages are sent to a participant after completing at least 10 minutes of MVPA to prompt further reflection on their experiences with exercise and experimenting with the Reflect strategy, shown in the Self-Experimentation to Cultivate Personalized Knowledge, Skills, and Practice section (Figure 5).

### Active Control

In addition to all the common elements described in the previous sections (eg, Fitbit smartwatch, US $50 in incentives for meeting step goals, and tailored SMS text messaging support), participants assigned to the active control group, similarly to those assigned to the YourMove intervention group, are instructed to engage in their normal level of PA for 1 week to establish a baseline, shown in Figure 7. This is followed by the intervention phase, during which participants receive a daily step goal along with an accompanying number of points earned each day that they reach their goal. Unlike the YourMove intervention, the daily step goal and accompanying number of points is kept fixed (10,000 steps per day and 150 points per day, respectively). The goal is delivered to participants via the standard Fitbit app features, and points are communicated via email. Participants also receive a weekly PA goal and the same accompanying motivational and informative SMS text messages as those for the YourMove intervention group described previously. Finally, the active control condition does not include the self-guided self-experimentation tool (Reflect) featured in the YourMove intervention condition.

### Data Management and Quality Assurance

Most study data will be collected, managed, and stored using the secure web-based tool REDCap hosted at UCSD [75]. REDCap provides an interface for data entry and storage, audit trails for tracking data input and changes, automated export procedures for data downloads to common statistical packages, and procedures for importing data from external sources. Furthermore, data are deidentified using a unique trial identification number assigned to each participant and kept confidential.

### Clinical Trial Integrity Practices While Accounting for the Need to Tune the Controller

As described in the Phase 3: Controller section, controller tuning is a common practice within control systems engineering and involves refining the controller for targets such as performance and robustness based on real-world observation. In this case, the key focus was on controller robustness. Robustness focuses on ensuring that the controller is programmed to be able to offer appropriate response options to the diversity of responses of those who will use the controller. Robustness is a critical target within control systems engineering. Critically, the tuning work focuses on refining how the controller responds to all participants, with any tuning changes made across all participants. Controller tuning is a common strategy used in control systems engineering and, thus, its lack of inclusion would result in an inappropriately tested control systems–driven behavioral intervention (ie, it would not reflect an appropriately operationalized COT approach). This is because, if controller tuning were excluded, the control system that is implemented would not be a rigorous implementation of standard procedures used in control systems engineering.

Controller tuning is not a normative requirement within traditional clinical trial work. Therefore, the team needed to develop an approach to guaranteeing clinical trial integrity to ensure the trustworthiness of the results of the RCT while simultaneously enabling the control systems team to engage in the required “tuning” work to ensure that we followed best practices from control systems engineering. Thus, the team developed the following sets of rules and procedures for data monitoring and fidelity that were reviewed and approved by the full study team (ie, all coauthors on this paper) before any data were collected.

At a high level, from the perspective of the RCT, control systems tuning was treated as part of the overall intervention package. With this stated explicitly, it is critical to note that, while the system we developed is scalable and automated, it does require a human to actively monitor the system to engage in tuning. We referred to this role as the “digital health PA tuning coach” described previously. From a scalability perspective, we assumed that any digital health implementation of this would include a staff member with the requisite data science and control systems expertise to fulfill this role on any scaled platform. Thus, this digital health PA tuning coach role, which was fulfilled by one of our team members conducting this set of activities as a part-time set of their total tasks, is another key element of the YourMove intervention that would need to be considered for any future implementation. On the basis of common practices in digital health and digital therapeutics companies, we postulate that it is a fair assumption that any digital health tool requires the right staffing to implement it at scale.

With the assumption that it is reasonable that data science expertise capable of tuning the controller will be present in a digital therapeutics company implementing our team’s approach, our team developed protocols to enable this digital health PA tuning coach role to be involved while not compromising the integrity of the clinical trial. Again, at a high level, a priori plans for maintaining clinical trial integrity included a clear separation of team personnel among (1) those who gather primary and secondary outcome data; (2) our study statistician and those conducting the primary analyses for the clinical trial; (3) those managing the study participants and, thus, who have awareness of group allocation by necessity of the behavioral trial design; and (4) the control systems engineering team tasked with implementing the standard practice of tuning the controller during the trial.

The following procedures for ensuring clinical trial integrity were established. First, eligible participants were randomly allocated to 1 of 2 conditions, YourMove or active control. The study statistician, without knowledge of participant characteristics, created a priori a computer-generated list involving block randomization accounting for sex as a biological variable. This randomization was used to assign participants to study conditions. Second, allocation concealment has been and will be maintained via the staff gathering clinical trial measurements, including the primary outcome measure of ActiGraph-assessed MVPA and physiological secondary laboratory-based outcomes (eg, BMI and submaximal oxygen consumption), remaining blinded to group allocation. Thus, the principal investigator (PI; EH) did not and will not have access to primary and secondary outcome data and will remain blinded to group allocation. Furthermore, the study statistician and all coinvestigators have remained and will remain blinded to primary and secondary outcome data while they are being gathered. Third, no data cleaning or analyses of primary and secondary laboratory-based outcomes have taken place or are planned until study completion save for basic data quality checks conducted by those who gathered the primary and secondary outcome data. No group comparisons, including possible comparisons with Fitbit or other data, are planned nor will be conducted until study completion. Group allocation will be revealed only after the primary analysis is completed.

Regarding enabling controller tuning while maintaining clinical trial integrity, the team responsible for controller tuning had no access to primary and secondary outcome data; the team tasked with tuning the controller were and will be unable to influence acquisition of the primary and secondary outcome data; study staff responsible for implementing the randomization procedure and, thus, aware of group allocation were not and will not be included in controller-tuning activities, remaining blind to those activities; the team responsible for tuning will not be able to run any analyses comparing differences between randomized groups; all tuning will use idiographic or N-of-1 analyses used as part of standard control systems engineering techniques for tuning a controller using data that the controller uses to make decisions, including steps per day as measured via Fitbit, EMA data, and weather data; a coinvestigator (PK) will be involved in all tuning discussions and provide behavioral insights to guide the tuning decision-making process, with final decision-making on the controller conducted by the multiple PI (DER); and the study PI (EH) will only take part in a limited matter regarding controller tuning. Specifically, the PI (EH) will only collaborate with the control systems engineering team during the final stages of publishing the tuning activities. These publications will report on only a selected subset of analyses conducted ideographically. These will be selected by the tuning team specifically because of their use in tuning the controller. Any results on tuning will not include results from all participants in the intervention condition to reduce possible risk of creating a bias regarding the integrity of the clinical trial. No analyses will be conducted within the control group dataset until after completion of the clinical trial.

### Statistical Analysis

#### Overview

For the comparison of a 12-month change in minutes per week of MVPA from baseline between the intervention and control groups, a 2-sided, 2-sample 2-tailed *t* test with a significance level of .05 (or a nonparametric approach such as the Wilcoxon rank sum test if parametric assumptions fail) will be conducted. In general, analyses will incorporate the intention-to-treat principle, which includes all participants who are randomized in the analysis. A mixed model for repeated measures (MMRM) will also be considered. The dependent variable in the MMRM is the change from baseline in minutes per week of MVPA at each postbaseline visit (6 and 12 months). Independent variables in the MMRM include study arm, visit (as a categorical variable), arm-by-visit interaction, minutes per week of MVPA at baseline, and potential covariates of interest (eg, baseline BMI, sex, age, ethnicity and race, marital status, parental status, employment status, and smoking status) that are determined a priori. Potential covariates significantly associated with outcome (*P*<.10) will be included in a multivariable logistic regression model. To avoid inflation of the type I error, we will use permutation tests for inference. Missing data will be evaluated. If the missingness is not random (nonignorable), multiple imputation or propensity weighting will be considered. There will be no planned interim analyses for efficacy or futility conducted for this study. In addition, a frequency table comparing the arms in terms of meeting the guidelines (dichotomized as *yes* or *no*) will be created, and a Fisher exact test will be conducted. Furthermore, a generalized estimating equations regression will be conducted with meeting the guidelines (*yes* or *no*) as the dependent variable and visit, arm, and visit by arm as interaction terms in the model using an appropriate correlation structure adjusted by similar covariates to those of the primary aim. Statistical analyses will be conducted using the statistical software R (version 4.1.2).

#### Power Considerations

Our sample size (N=386) provides sufficient power for our primary aim. On the basis of a 2-sided, 2-sample *t* test, with a sample size of 193 in each arm (386 participants in total) and a type I error (α) of .05, we achieve 80% power to detect a standardized effect size [1-3,8,9] of 0.32 for comparing a month 12 change from baseline in minutes per week of MVPA between arms, accounting for an attrition rate of 20% in measurement (ie, completing the ActiGraph measurement battery at 12 months but not necessarily adhering to the use of the DHI; Table 2). We have powered for a 0.32 effect size, through which we anticipate a change from baseline of 45 minutes of MVPA after 12 months in the control arm and a 75-minute change from baseline in MVPA for the intervention arm assuming a pooled SD of change of 94 minutes and a mean change score difference of 30 minutes per week of MVPA.

**Table 2:** Sample size and power (2-sided α=.05).

Power	Effect size	Sample size per group	Overall sample size	Overall sample size considering 15% attrition	Overall sample size considering 20% attrition
0.8	0.32	154	308	362	386
0.8	0.36	122	244	288	306
0.8	0.4	99	198	234	248
0.85	0.32	176	352	414	440
0.85	0.36	139	278	328	348
0.85	0.4	113	226	266	284

To develop this power calculation, we examined effect sizes from 3 different studies available at the time of study design [26] plus our own previous formative research results [93,95-98] to establish an effect size of 0.32 as reasonable. When combined, previous studies and formative research results address power considerations related to using similar measurement batteries, detecting meaningful effects at 12 months between 2 active interventions and plausibly meaningful effects to be anticipated when comparing our YourMove intervention approach to our active control. First, these estimates are consistent with those of the study by Lynch et al [98], which we used as a reference as it conforms to effects that we observed in our formative research. It evaluated a technology-based intervention and used a similar measurement protocol, particularly an ActiGraph monitor, to assess minutes per week of MVPA. That said, the trial did not assess differences at 12 months, nor did it compare 2 active interventions. As such, we used the Community Health Advice by Telephone trial as a second reference, whereby an effect size of 0.32 (ie, the same effect size as that for our study) was also observed as the difference between 2 active and successful PA interventions at 12 and 18 months after baseline, with one being a technology-based intervention and the second being a human-based intervention [97,99]. As our RCT includes an active goal-setting intervention, we also examined effect sizes in a third trial by previous studies [95,96]. Specifically, Adams et al [95] conducted a trial comparing adaptive step goals to static step goals (ie, 10,000 steps per day) and also immediate reward provision versus delayed reward provision (with both groups, similarly to ours, receiving gift cards if they met a daily step goal target). Results indicated that all conditions significantly increased by, on average, 2389 steps per day over 4 months (which aligns with our pilot study of JustWalk version 1 of 2651 steps per day [100] at 3 months). Results further indicated a significant difference at intervention completion favoring immediate over delayed reward (with immediate reward in alignment with our protocol) and an effect size of 0.32 comparing these 2 active interventions. The condition most similar to our intervention condition (ie, adaptive goals+immediate feedback) exhibited gradual increases in steps across the intervention, including until study end, which supports our assertion of the value of gradual habit formation. In our pilot study, we had 5% attrition over 3 months, and the study by Adams et al [95] had an attrition rate of 11% at 4 months. On the basis of this, our oversampling to support up to 20% attrition at 12 months is justifiable, particularly when considering the use of the ActiGraph monitor for assessment instead of using a measurement approach that is linked to the intervention in some way. In summary, based on the overall state of the science, being able to detect an effect size of 0.32, which equates to a change difference of 30 minutes per week of MVPA between YourMove and the active control via ActiGraph at 12 months, would illustrate both a clinically meaningful difference and also a difference that is conservative enough to be observable between 2 active interventions based on prior work.

## Results

The YourMove study was funded in July 2020 and received institutional review board approval on July 1, 2020. Recruitment and enrollment began in October 2022, and a total of 386 participants were enrolled by August 2024. Data collection is ongoing and expected to conclude by August 2025. Data analysis has not yet begun, and results are anticipated in early 2026.

## Discussion

### Overview

Our prior work has emphasized the need for personalized interventions that can adapt dynamically to each individual’s evolving context and behavioral patterns. We observed that internal factors (eg, stress and busyness) and external factors (eg, the weather and day of the week) influence PA in ways that vary not only across individuals but also within individuals over time. These findings align with and extend previous research showing that DHIs for PA tend to yield moderate short-term effects and are generally acceptable, yet their benefits often diminish over time and are typically limited to specific subpopulations. Together, this evidence underscores a critical gap in current intervention design—the need for approaches that are responsive to the inherently dynamic and idiosyncratic nature of PA rather than relying on static behavior change assumptions.

To address this gap, the YourMove DHI integrates two core components: (1) habit formation mechanisms driven by the team’s proposed COT approach and (2) knowledge, skill, and practice development components via a self-experimentation approach modeled after the Schraefel et al [48] experiment in a box (XB) approach to improve MVPA. There are many potential contributions of this work. First, our intervention is the first scaled deployment and testing of the team’s overall COT approach, described previously. Designed to be highly scalable for improving behaviors and reducing chronic disease risk, if effective, it could be used widely through smartwatch and smartphone technology. In addition, this study will be a rigorous evaluation of the COT approach via an RCT. If our approach to offering personalized and perpetually adaptive support is helpful for PA, it will provide justification for extending the approach to other health behaviors that are dynamic and idiosyncratic, such as weight loss or addiction.

### Previous Research That Informed Our COT Element

As a proof of concept for the COT approach used in YourMove, system ID techniques were used to develop an idiographic dynamic model using participant data from the team’s previous study, Just Walk [89], which included an intervention aimed at promoting walking behavior among sedentary adults. While YourMove builds on foundational insights from Just Walk, it represents a deliberate and planned expansion of the original work. Notably, this protocol includes a broader age range (adults aged ≥25 years) and is not limited to individuals who are overweight or obese, as was emphasized in earlier protocol versions and peer-reviewed materials. These changes were implemented in response to iterative design work that was explicitly outlined in the funding grant and intended to enhance generalizability and future scalability. In addition, while both studies center on mobile health–based PA promotion, YourMove incorporates new components, including a knowledge, skill, and practice self-experimentation element and broader COT infrastructure to support real-time N-of-1 adaptation that is both guided by the controller and self-guided. These deviations from the original protocol are intended to enhance generalizability and reflect iterative improvements informed by the design work that was part of the funding grant.

On the basis of this formative work, we conducted a number of analyses intended to lead to more predictive models that informed intervention development and decision policies for a personalized and perpetually adapting intervention. The estimated model was subsequently used to evaluate a control-oriented decision-making algorithm designed for an adaptive DHI that promotes PA in sedentary adults within a simulation environment [89]. The decision-making framework was based on 3DoF-KF HMPC formulation, incorporating a mixed logical dynamic framework to represent categorical and logical decisions within the policy. A three-degree-of-freedom tuning approach was implemented, allowing for independent adjustments for response speed to set point tracking and disturbance rejection, both measured and unmeasured. Simulation results highlighted the integration of system ID and the HMPC controller formulation, demonstrating the potential for optimal and personalized PA interventions—a crucial step toward large-scale dissemination of behavioral interventions for PA promotion. The estimated model displayed strong predictive capabilities while maintaining a good fit to the data. This model was used as the predictive component in simulations to assess the efficacy of the 3DoF-KF HMPC framework in delivering individualized PA interventions. Simulation outcomes showed the HMPC algorithm to be highly effective in guiding personalized PA interventions. In addition, within the HMPC algorithm, the controller was reconfigured to support a maintenance phase, reducing or eliminating financial incentives to help participants sustain healthy PA levels without dependence on rewards [48].

Thus, the YourMove study described in this protocol is the logical next step in this systematic line of research meant to develop ways to use control systems engineering methods to guide the creation of personalized and perpetually adaptive interventions. The results of this trial will provide a robust nomothetic test of the value of the many decisions and efforts of the team to create a robust operationalization of this type of personalized and perpetually adapting intervention. Significant results suggesting improvements in MVPA relative to an active control will provide compelling evidence for both digital health and digital therapeutics companies to consider incorporating this algorithmic approach into their tools. In addition, these results will provide justification for the continued development and expansion of the use of control systems engineering approaches for providing highly personalized and perpetually adaptive interventions for individuals, thus filling a critical gap in care, namely, the capacity to provide the “right” type of support that is appropriate for each person over time.

### Previous Research Informing Our Self-Experimentation Tool

The Reflect tool was based on the XB framework developed by Schraefel et al [48]. Contrary to common digital health tools that seek to provide the solutions for participants, the XB approach allows participants to experiment with general health heuristics to discover what is useful for themselves. Through self-experimentation, an individual can build knowledge, skills, and practice to transform a general health heuristic (eg, national guidelines for PA) into a personal health heuristic (eg, a regular exercise routine that adapts to different contexts) [48]. Aligned with self-determination theory, the XB framework gives a participant the opportunity to develop autonomy to determine and use strategies that work for them without pressure to adopt ones that are not useful [100]. In this way, participants are the agents of achieving desired behavior change rather than the intervention or technology itself.

This work also aligns with other frameworks of self-experimentation such as the personal science framework [101] and self-study [102]. Personal science is illustrated by a cycle of five activities in which individuals examine their personal health by (1) *asking questions* unique to their health status and behaviors; (2) *designing* to discover and apply necessary changes; (3) *observing* to self-track meaningful data; (4) *reasoning* to reassess questions, designs, and observations; and (5) *discovering* what is helpful or needs further questioning [101]. Similarly, self-study involves experimentation and tracking at an individual level to establish autonomy and make meaningful impacts on one’s health [48]. By using the XB and personal science frameworks and self-study, Reflect is a tool in the YourMove intervention designed to promote intrinsic discovery of personal PA health heuristics to enable resilient PA practice.

While this intervention component was a secondary element to the overall intervention, if our trial results suggest significant differences in our active intervention relative to the control, it will provide empirical justification for continued study and exploration on ways in which self-experimentation approaches can be developed within digital health and digital therapeutics tools. We see particular value in this approach as a strategy for ensuring the ethical use of these digital technologies via an explicit incorporation of the person determining whether an intervention is working for them based on their own self-experiments.

### Study Limitations

Several limitations inherent to RCTs on behavioral interventions should be acknowledged. First, due to the nature of digital behavioral interventions, observer bias may have arisen as it is challenging to achieve full blinding in these types of studies. Second, the primary outcomes were measured at discrete time points, providing only snapshots of behavior change over the study period. This approach may lack sensitivity to capture the dynamic, continuous changes in behavior that occur within individuals (although the team can overcome this via the use of Fitbit data as a secondary check). Third, this study did not use a fully random sampling approach for recruitment, which may limit the generalizability of the findings, although the approaches do conform to standard practices for behavioral interventions. Finally, as is the case with any RCT, there are potential limitations in internal validity, including potential unmeasured confounding variables and issues related to participant adherence, which are common in behavioral intervention trials.

### Conclusions

Overall, this work will examine whether the team’s hypothesized approach to creating a personalized and perpetually adaptive DHI can result in increased MVPA in comparison to an active control condition that is meant to control for common behavioral strategies (eg, self-monitoring, goal setting, financial incentives, and psychoeducation) while also mimicking common digital health worksite wellness offerings that were available and common when the study was being designed. This research marks an important step toward developing scalable solutions for sustainable, personalized, and perpetually adapting PA support.

## Appendix

Multimedia Appendix 1 YourMove Reflect tool for week 1.

Multimedia Appendix 2 YourMove Reflect tool week 1 summary email.

Multimedia Appendix 3 YourMove Reflect tool for the follow-up weeks.

Multimedia Appendix 4 YourMove Reflect tool summary email for the follow-up weeks.

Multimedia Appendix 5 YourMove Reflect tool experiment strategies.

Multimedia Appendix 6 Reflect tool week 1 survey video.

Multimedia Appendix 7 Reflect tool survey video for the follow-up weeks.

Multimedia Appendix 8 Weekly schedule and examples of SMS text messages delivered in the YourMove intervention.

Multimedia Appendix 9 Peer-review report from PRDP - Psychosocial Risk and Disease Prevention Study Section (National Institutes of Health, USA).

## Abbreviations

BCT: behavior change technique
COT: control optimization trial
DHI: digital health intervention
EMA: ecological momentary assessment
EPARC: Exercise and Physical Activity Resource Center
HMPC: hybrid model predictive control
MMRM: mixed model for repeated measures
MVPA: moderate to vigorous physical activity
PA: physical activity
PAR-Q: Physical Activity Readiness Questionnaire–Revised
PI: principal investigator
RCT: randomized controlled trial
REDCap: Research Electronic Data Capture
SCT: social cognitive theory
UCSD: University of California, San Diego
XB: experiment in a box

## Data Availability

The datasets generated or analyzed during this study are available from the corresponding author on reasonable request.
