# Supplementary material for: Optimizing and Testing an Individualized and Adaptive Physical Activity Digital Health Intervention: Protocol for a Control Optimization Trial Embedded Within a Randomized Controlled Trial
Source: JMIR Res Protoc. 2025 Aug 15;14:e70599. doi: 10.2196/70599 (PMC12397713; doi:10.2196/70599)
Supplement: Multimedia Appendix 1 [file resprot_v14i1e70599_app1.pdf]

YourMove Reflect

INTRO MODULES

Welcome to YourMove and your personal exercise experimentation tool - we're calling it Reflect!

Create videos that move people to action Make a video

Reflect is designed to help you figure out strategies to develop a regular exercise routine. There are a lot of great strategies out there, but they don't work for everyone. And that's why **Reflect** exists.

Reflect works like this: First, we'll **offer strategies** that have helped others exercise regularly. Next, you'll **pick one to try out** for a week. Then, we'll check in on **how the strategy is working for you** on your Fitbit with messages like, "Did you try a strategy from **Reflect** this time?" and "Did the **Reflect** strategy help?" No matter what happens, you'll have learned something important.
If it helped, great! Keep doing it! If it didn't help, also great! **You learned something.** Last, each week we'll remind you to come back to **Reflect** to try the same strategy again or try something new.

Why is Reflect 12 months long? It's long enough to support you through all seasons, but short enough to prevent you from becoming reliant on it. This way **you can figure out strategies that work for every part of the year** while learning skills of self-reliance and problem solving.

After 12 months, we hope you'll learn THREE things:
1. **Which strategies help** you exercise
2. How to use experimentation to **create other resilient, healthy routines**
3. How to use the strategies and **Reflect** approach to **roll with all the changes life throws at you**

Going into this for the first time just remember that **Reflect is about helping YOU reach YOUR exercise goals**. Trust yourself and know that finding what works takes time. *Time to get started!*

OVERALL EXERCISE PLANNING_1

This week, you will start getting text messages with your weekly MVPA goal, tips to get active, and your progress meeting your goals. As a reminder, MVPA stands for moderate to vigorous physical activity and is tracked via your Fitbit through "active zone minutes".

While your text messages are there to keep you on track with your exercise goals, **Reflect** will help you to plan out exactly HOW you'll reach your goals. Each time you complete **Reflect** you'll be able to adjust your plan to match your schedule and fitness level in a way that works best for you.

To get started, within your weekly MVPA goal how would you like to get in your active minutes? Through more moderate vs. vigorous intensity exercises?

***Moderate intensity** = able to talk between breaths (brisk walking, hiking, dancing, swimming/water aerobics, golf, etc.)*
***Vigorous intensity** = difficult to talk without pausing (running, cycling, elliptical, aerobics class, basketball, etc.)*

- mostly moderate-intensity activity only
- an even mixture of moderate and vigorous activity
- mostly vigorous intensity activity only

Got it! Now let's nail down what type of exercise you'd like to try by learning what you've done in the past.

Start by thinking about any sort of exercise you've enjoyed and/or type(s) of exercise you've wanted to try and think you might enjoy. Nothing coming to mind? We suggest you start simply and select something like walking (for moderate activity) or running/treadmill (for vigorous activity).

First, select the one you are MOST interested in trying this week (you will be able to select up to 2 more)

- biking outdoors
- bootcamp
- circuit training
- elliptical
- playing golf
- hiking
- interval workout
- kickboxing
- martial arts
- running outdoors
- spinning indoors
- stair climber
- swimming
- playing tennis
- treadmill indoors
- walking

You chose **${q://QID29/ChoiceGroup/SelectedChoices}**
Want to add another exercise to try?
Now is great time to experiment with new exercises. You won't know if you like it or not unless you try!

- No - I'm just going to focus on the exercise above
- » biking outdoors
- » bootcamp
- » circuit training
- » elliptical
- » playing golf
- » hiking
- » interval workout
- » kickboxing
- » martial arts
- » running outdoors
- » spinning indoors
- » stair climber
- » swimming
- » playing tennis
- » treadmill indoors
- » walking

You've chosen **${q://QID29/ChoiceGroup/SelectedChoices}**
and **${q://QID32/ChoiceGroup/SelectedChoices}**
Want to add one more exercise to try?
Now is great time to experiment with new exercises. You won't know if you like it or not unless you try!

- » No - I'm just going to focus on the exercise above
- » biking outdoors
- » bootcamp
- » circuit training

- » elliptical
- » playing golf
- » hiking
- » interval workout
- » kickboxing
- » martial arts
- » running outdoors
- » spinning indoors
- » stair climber
- » swimming
- » playing tennis
- » treadmill indoors
- » walking

OVERALL EXERCISE PLANNING_2

You selected **${e://Field/Exercise_all}**.

For this first week, rather than worry about whether you're getting enough active minutes, simply try out what you selected to see what you like. If you like one (or more) of your choices, great! Stick with it. If you don't like what you chose, also great! You can try something else next week.

This is a learning experience and takes time. Down the line when you find an activity or two that works for you, we'll start building up your active minutes.

Now that you've landed on what you'd like to do for exercise, it's time to experiment! Each of the strategy categories listed below will help to build a regular exercise routine. As a reminder, success here is completely defined by you. Much like discovering what types of exercise will work for you, finding what strategies are helpful may take time. Trust yourself and use **Reflect** to continue experimenting until you find the best fit.

To get started, think through what kinds of strategies would be most beneficial to work on.

- *Not sure to where to begin?* Perhaps select strategies to help you fit exercise into your life.
- *Is exercise something you've found unenjoyable?* Strategies to help increase your enjoyment in exercising could be helpful.
- *Curious to see how exercise might impact your overall mood and energy?* Give strategies to reduce negative feelings through exercise a go.
- *Find exercise to be uncomfortable?* Maybe explore strategies to reduce your sense of discomfort while exercising.

Which are you most curious and interested in right now? There is no wrong answer. Go with your gut and pick the one you feel drawn to. If it doesn't work out - no problem! Just come back and try one of the other ones later.

- ◯ Strategies to help you fit exercise into your life
- ◯ Strategies to help increase your enjoyment in exercising
- ◯ Strategies to reduce negative feelings through exercise
- ◯ Strategies to reduce your sense of discomfort while exercising

DEVELOPING PLANS MODULES_1

Alright! So, you want to experiment with strategies to help you fit exercise into your life. We've got a ton of them for you to try.

Hover over each option and read through what each entails. Then, **pick one** that sounds the most appealing, helpful, and/or interesting to you. (Don't worry, you'll have plenty of chances to try out more in the future!)

Keep in mind that it's OK if you select one that sounds good now but, after you try it, it doesn't work for you. You are learning, which is what matters most. You are always able to come back and try something else!

Hover over the text to see a description of the practice.

- ◯ Prep the night before
- ◯ Have a "plan c"

- ○ Start with 10
- ○ Make a date with exercise
- ○ Seize the morning
- ○ A sweat a day
- ○ Workout with a buddy
- ○ Reward yourself!
- ○ Create visual reminders
- ○ Stick to a 2-day rule
- ○ Never miss a Monday
- ○ Exercise in short bursts
- ○ Exercise shoe trick
- ○ Be a weekend warrior

DEVELOPING PLANS MODULES_2

Cool! You selected
${e://Field/Heuristic}
to experiment with this week. The goal of this is to help you to fit in your exercise selections in your life and, ultimately, reach your weekly MVPA goals.

Specifically, this strategy asks that you **${e://Field/Heuristic_description}**

If the strategy you selected doesn't end up working for you, that's OK! Not every strategy will work for everyone and it can take some trial-and-error to find what works for you and your unique circumstances. Next time you check in with **Reflect** you can select something else.

Your plan this week is to experiment with strategies to increase your enjoyment in exercising. Great choice! Because if you learn to love exercise, you'll feel more driven to do it, particularly even if you feel tired or are not in the mood.

Start by reviewing the following positive experiences people often link with exercise. Hover over each one for a more detailed description. Which ones would also be a positive experience for you?

Next, **pick one** you can experiment with while exercising this week. If there is something you would want to try but it's not listed, select "Other" and specify.

Hover over the text to see a description of the practice.

- ○ Listening to a podcast/audiobook
- ○ Listening to music
- ○ Watching TV
- ○ Enjoying the outdoors
- ○ Enjoying the weather
- ○ Getting some alone time
- ○ Socializing with others
- ○ Enjoying an active hobby
- ○ Being able to Decompress/Unwind
- ○ [ ] Other

DEVELOPING PLANS MODULES_3

Nice! You're going to try to

${e://Field/Enjoyment}

when you exercise this week. ${e://Field/Enjoyment_description}.

While experimenting, notice if what you selected makes exercise more enjoyable. If it does, wonderful! You're a step closer in making exercise a positive experience. If it doesn't, no problem! You learned something and are free to find what *does* work for you. And, remember, you are able to return to **Reflect** and explore alternatives.

Nice choice! You are going to try out strategies to reduce negative feelings through exercise. Exercise impacts both physical *and* mental health. This strategy is a good way to experiment with how negative feelings you may experience change with exercise and if that ultimately encourages you to move more.

Review each of the negative feelings below and think about which ones may be impacted by exercise for you. Then, **select one** that resonates most. If there is something you'd like to add, select "Other" and specify.

- feeling anxious
- feeling stressed
- feeling sad/depressed
- feeling fatigued
- feeling discouraged
- feeling like you're not living up to identity as an exerciser
- feeling physical aches and pains
- feelings associated with a particular illness or condition you have (Type the illness/condition below) ________
- ________ Other negative feelings

Alright! So, this week you are going to focus on your experience with

${q://QID15/ChoiceGroup/SelectedChoicesTextEntry}

before, during, and after you exercise. Think about how what you selected changes (or doesn't) over time - this is no "right" experience with this.

If you find that there is an improvement, fantastic! Exercise could be seen as a healthy way to cope with this negative feeling. If you find no change or that your feelings worsen, it's OK. You've discovered something about yourself. You can come back to **Reflect** and experiment with another strategy that will be more helpful.

OK, so you are wanting to experiment with strategies to reduce experiences of discomfort while exercising.

There are a lot of experiences that come up during exercise that can be seen as negative. Look over what we've listed below and think about which you find to be uncomfortable, unpleasant, discouraging, etc. when you exercise. Next, **select one** that stands out to you the most.

- ○ Soreness 😫
- ○ Fatigue 😴
- ○ Feeling hot 🥵
- ○ Feeling sweaty 😰
- ○ Not being able to perform well 😔
- ○ Apathy 🥱

DEVELOPING PLANS MODULES_4

OK, so **${e://Field/Discomfort}** is something that comes up for you as a negative experience when you exercise. Changing how we think about these kinds of experiences can help to reduce barriers to exercise and encourage us to exercise regularly.

This week, when you experience ${e://Field/Discomfort} during exercise, we want you to try and reframe it as a neutral or even positive experience. For example, for ${e://Field/Discomfort} you could reframe it as ${e://Field/Discomfort_reframe}.

If you find that rethinking this experience in a neutral or positive way works for you, amazing! If you find that it doesn't, give it a few chances and always know you can come back to **Reflect** and try another strategy that may be more helpful for you.

Summary Module

Let's recap! To reach your MVPA goal this week you are:

- Aiming to get your active minutes through **${e://Field/MVPA_ratio}.**
- Focusing on trying **${e://Field/Exercise_all}** as often as you can this week for aerobic exercise.
- Experimenting with **${e://Field/Strategy}** as a way to help you to exercise regularly.

We'll check in with you each time you exercise this week via your Fitbit to see how your experiment is going. So when you see a message asking you about **Reflect** we're seeing if **${e://Field/Summary_description}**. Check your watch and Fitbit app daily to see how your active minutes stack up and how the moderate-vigorous intensity ratio and the exercises you selected are working for you.

Especially in these first few weeks of YourMove, take the pressure out of it! Simply see this week as a chance to begin to learn what might be helpful for you as you start to establish an exercise routine. Jot down your experiences - good and bad - in a place that is convenient and easy to remember, like your planner or on your phone. This way you can refer to them during next week's check-in.

UCSD IRB# 200733

Powered by Qualtrics
