# Supplementary material for: Optimizing and Testing an Individualized and Adaptive Physical Activity Digital Health Intervention: Protocol for a Control Optimization Trial Embedded Within a Randomized Controlled Trial
Source: JMIR Res Protoc. 2025 Aug 15;14:e70599. doi: 10.2196/70599 (PMC12397713; doi:10.2196/70599)
Supplement: Multimedia Appendix 3 [file resprot_v14i1e70599_app3.pdf]

YourMove Reflect

Intro Block

Welcome back to **Reflect**! We'll start by checking in on how you wanted to go about meeting your MVPA goal from last week and what you'd like to do to meet your goal this week. Then, we'll see how experimenting with ${e://Field/Strategy} went for you. Lastly, you'll decide what you want to experiment with this week as you continue to establish an exercise routine. Let's get started.

Would you like to revisit our informational video before you get started?

- ○ Yes
- ○ No

Create videos that move people to action Make a video

MVPA Ratio

It's now time to see how well your exercise plan worked for you this week. Keep in mind, any feedback helpful. Adopting and sticking to an exercise plan is something that takes time to do. So if you nailed your plan for the week, great! You may have found something that works well for you. And if your plan didn't quite pan out, that's also OK! It's simply a sign to switch it up and find a better fit.

Last time you stated that you want to reach your weekly MVPA goal through **${e://Field/MVPA_ratio}**. Is this still the case?

- Yes
- No

OK! Let's change that. Now within your weekly MVPA goal how would you like to get in your active minutes? Through more moderate vs. vigorous intensity exercises?

***Moderate intensity** = able to talk between breaths (brisk walking, hiking, dancing, swimming/water aerobics, golf, etc.)*
***Vigorous intensity** = difficult to talk without pausing (running, cycling, elliptical, aerobics class, basketball, etc.)*

- mostly moderate-intensity activity only
- an even mixture of moderate and vigorous activity
- mostly vigorous intensity activity only

XP - Ex Count = 1

Last week, you wanted to try **${e://Field/Exercise_1}** for aerobic exercise.
Did you get a chance to try it last week?

- Yes
- No

Great! How did you like **${e://Field/Exercise_1}**?

- Not at all
- Not so much
- Kind of
- A good amount
- A lot

This is great to hear! Let's stick with **${e://Field/Exercise_1}** again this week as doing something you enjoy for exercise helps to keep you consistent and motivated.

That's good to recognize that you weren't really a fan of **${e://Field/Exercise_1}** and may be a sign that it's time to try something else - because if something isn't enjoyable, we're less likely to stick with it.

Let's give something else a go this week - perhaps another activity will be a better fit for you.

No problem - sometimes its hard to find time to try a new exercise.

Do you want to try **${e://Field/Exercise_1}** again this week?

- Yes
- No

XP - Ex Count = 2

Last week, you wanted to try **${e://Field/Exercise_all}** for aerobic exercise.

Now, we'll ask if you tried those exercises and whether or not you liked them.

Did you get a chance to try **${e://Field/Exercise_1}** last week?

- Yes
- No

Great! How did you like **${e://Field/Exercise_1}**?

- Not at all
- Not so much
- Kind of
- A good amount
- A lot

This is great to hear! Let's stick with **${e://Field/Exercise_1}** again this week as doing something you enjoy for exercise helps to keep you consistent and motivated.

That's good to recognize that you weren't really a fan of **${e://Field/Exercise_1}** and may be a sign that it's time to try something else - because if something isn't enjoyable, we're less likely to stick with it.

Let's give something else a go this week - perhaps another activity will be a better fit for you.

No problem - sometimes its hard to find time to try a new exercise.

Do you want to try **${e://Field/Exercise_1}** again this week?

- Yes
- No

Did you get a chance to try **${e://Field/Exercise_2}** last week?

- Yes
- No

Great! How did you like **${e://Field/Exercise_2}**?

- Not at all
- Not so much
- Kind of
- A good amount
- A lot

This is great to hear! Let's stick with **${e://Field/Exercise_2}** again this week as doing something you enjoy for exercise helps to keep you consistent and motivated.

That's good to recognize that you weren't really a fan of **${e://Field/Exercise_2}** and may be a sign that it's time to try something else - because if something isn't enjoyable, we're less likely to stick with it.

Let's give something else a go this week - perhaps another activity will be a better fit for you.

No problem - sometimes its hard to find time to try a new exercise.

Do you want to try **${e://Field/Exercise_2}** again this week?

- Yes
- No

XP - Ex Count = 3

Last week, you wanted to try **${e://Field/Exercise_all}** for aerobic exercise.

Now, we'll ask if you tried those exercises and whether or not you liked them.

Did you get a chance to try **${e://Field/Exercise_1}** last week?

- Yes
- No

Great! How did you like **${e://Field/Exercise_1}**?

- Not at all
- Not so much
- Kind of
- A good amount
- A lot

This is great to hear! Let's stick with **${e://Field/Exercise_1}** again this week as doing something you enjoy for exercise helps to keep you consistent and motivated.

That's good to recognize that you weren't really a fan of **${e://Field/Exercise_1}** and may be a sign that it's time to try something else - because if something isn't enjoyable, we're less likely to stick with it.

Let's give something else a go this week - perhaps another activity will be a better fit for you.

No problem - sometimes its hard to find time to try a new exercise.

Do you want to try **${e://Field/Exercise_1}** again this week?

- Yes
- No

Did you get a chance to try **${e://Field/Exercise_2}** last week?

- Yes
- No

Great! How did you like **${e://Field/Exercise_2}**?

- Not at all
- Not so much
- Kind of
- A good amount
- A lot

This is great to hear! Let's stick with **${e://Field/Exercise_2}** again this week as doing something you enjoy for exercise helps to keep you consistent and motivated.

That's good to recognize that you weren't really a fan of **${e://Field/Exercise_2}** and may be a sign that it's time to try something else - because if something isn't enjoyable, we're less likely to stick with it.

Let's give something else a go this week - perhaps another activity will be a better fit for you.

No problem - sometimes its hard to find time to try a new exercise.

Do you want to try **${e://Field/Exercise_2}** again this week?

- Yes
- No

Did you get a chance to try **${e://Field/Exercise_3}** last week?

- Yes
- No

Great! How did you like **${e://Field/Exercise_3}**?

- Not at all
- Not so much
- Kind of
- A good amount
- A lot

This is great to hear! Let's stick with **${e://Field/Exercise_3}** again this week as doing something you enjoy for exercise helps to keep you consistent and motivated.

That's good to recognize that you weren't really a fan of **${e://Field/Exercise_3}** and may be a sign that it's time to try something else - because if something isn't enjoyable, we're less likely to stick with it.

Let's give something else a go this week - perhaps another activity will be a better fit for you.

No problem - sometimes its hard to find time to try a new exercise.

Do you want to try **${e://Field/Exercise_3}** again this week?

- Yes
- No

Add New XP - Negative Only

Ok so you weren't really enjoying
${e://Field/Neg_ex_1}
${e://Field/Neg_ex_2}
${e://Field/Neg_ex_3}
lets find something new to do this week.

Select a new exercise that you are MOST interested in trying this week (you will be able to select up to 2 more)

- biking outdoors
- bootcamp
- circuit training
- elliptical
- playing golf
- hiking
- interval workout
- kickboxing
- martial arts
- running outdoors
- spinning indoors
- stair climber
- swimming

- playing tennis
- treadmill (indoors)
- walking

Great! You're going to try **${q://QID224/ChoiceGroup/SelectedChoices}**

Do you want to choose another exercise to try this week?
Remember, now is great time to experiment with new exercises. You won't know if you like it or not unless you try!

- No thanks - I'll just focus on the exercise above
- » biking outdoors
- » bootcamp
- » circuit training
- » elliptical
- » playing golf
- » hiking
- » interval workout
- » kickboxing
- » martial arts
- » running outdoors
- » spinning indoors
- » stair climber
- » swimming
- » playing tennis
- » treadmill (indoors)
- » walking

Awesome! You chose **${q://QID224/ChoiceGroup/SelectedChoices}**
and **${q://QID225/ChoiceGroup/SelectedChoices}**

Want to choose one more exercise to try this week?

Remember, now is great time to experiment with new exercises. You won't know if you like it or not unless you try!

- » No thanks - I'll just focus on the exercise above
- » biking outdoors
- » bootcamp
- » circuit training
- » elliptical
- » playing golf
- » hiking
- » interval workout
- » kickboxing
- » martial arts
- » running outdoors
- » spinning indoors
- » stair climber
- » swimming
- » playing tennis
- » treadmill (indoors)
- » walking

Add New XP - Positive w/ Negative

Ok good to hear you're going to keep trying **${e://Field/Pos_ex_all}** this week!

But, you weren't really enjoying or didn't get to try
${e://Field/Neg_ex_1}
${e://Field/Neg_ex_2}
${e://Field/Neg_ex_3}
lets find something new to do this week, if you'd like.

Do you want to add any new exercises to try this week or do you just want to stick with **${e://Field/Pos_ex_all}**?

Remember, now is great time to experiment with new exercises. You won't know if you like it or not unless you try!

- No - I'm just going to focus on the exercise above
- biking outdoors
- bootcamp
- circuit training
- elliptical
- playing golf
- hiking
- interval workout
- kickboxing
- martial arts
- running outdoors
- spinning indoors
- stair climber
- swimming
- playing tennis
- treadmill (indoors)
- walking

Great! You're going to keep trying **${e://Field/Pos_ex_all}** and add **${q://QID227/ChoiceGroup/SelectedChoices}**

Do you want to choose one more exercise to try this week?
Remember, now is great time to experiment with new exercises. You won't know if you like it or not unless you try!

- » No - I'm just going to focus on the exercise above
- » biking outdoors
- » bootcamp
- » circuit training
- » elliptical

- » playing golf
- » hiking
- » interval workout
- » kickboxing
- » martial arts
- » running outdoors
- » spinning indoors
- » stair climber
- » swimming
- » playing tennis
- » treadmill (indoors)
- » walking

Oops! Looks like you have duplicate exercises - please click the back button and choose different exercises.

Add New XP - Positive Only

WOW! Looks like you found a routine that works for you! Keep on enjoying **${e://Field/Pos_ex_all}** and we'll check in again next week.

Ok good to hear you're going to keep trying **${e://Field/Pos_ex_all}** this week!

Do you want to add any new exercises to try this week or do you just want to stick with **${e://Field/Pos_ex_all}**?
Remember, now is great time to experiment with new exercises. You won't know if you like it or not unless you try!

- No - I'm just going to focus on the exercise above
- biking outdoors
- bootcamp
- circuit training
- elliptical

- playing golf
- hiking
- interval workout
- kickboxing
- martial arts
- running outdoors
- spinning indoors
- stair climber
- swimming
- playing tennis
- treadmill (indoors)
- walking

Great! You're going to keep trying **${e://Field/Pos_ex_all}** and add **${q://QID230/ChoiceGroup/SelectedChoices}**

Do you want to choose one more exercise to try this week?
Remember, now is great time to experiment with new exercises. You won't know if you like it or not unless you try!

- » No - I'm just going to focus on the exercise above
- » biking outdoors
- » bootcamp
- » circuit training
- » elliptical
- » playing golf
- » hiking
- » interval workout
- » kickboxing
- » martial arts
- » running outdoors
- » spinning indoors

- » stair climber
- » swimming
- » playing tennis
- » treadmill (indoors)
- » walking

Oops! Looks like you have duplicate exercises - please click the back button and choose different exercises.

Exercise Summary

The plan for this week is to do **${e://Field/Exercise_all}** for aerobic exercise with the goal of getting 150+ minutes of moderate activity, 75+ minutes of vigorous activity, or a mixture of the two.

If you're having trouble finding time to exercise, it might be a good idea to add when you plan to exercise to the same place you keep track of your meetings, appointments, and other commitments so that it's just as much a priority.

Now, let's explore how your experiment with **${e://Field/Strategy}** went this past week.

STRATEGIES TO FIT EXERCISE CHECK IN

As a reminder, last week you selected **${e://Field/Heuristic}** as a strategy to help you fit exercise into your life.

Did you get a chance to try it out?

- Yes
- No

Great to hear you gave it a shot!

	Not at all	Not so much	Kind of	A good amount	A lot
How did you like it?	○	○	○	○	○
How helpful was it for you?	○	○	○	○	○
Did it fit into your life easily?	○	○	○	○	○

So it looks like although ${e://Field/Heuristic} wasn't something you were a big fan of, it did help you to exercise more and fit into your life easily. This is important to note, as it can help you to decide what you want to do in the weeks ahead.

Take a moment to think about your experience with the strategy this past week. Is it something you'd like to try again?

- ○ I'll give it a try again
- ○ I'd like to try something new

Good to know! It sounds like you were a big fan of ${e://Field/Heuristic}, but it wasn't very helpful in getting you to exercise more nor was it a good fit into your life.

Take a moment to think about your experience with the strategy this past week. Is it something you'd like to try again?

- ○ I'll give it a try again
- ○ I'd like to try something new

OK! So ${e://Field/Heuristic} was enjoyable and helpful, but not really a good fit into your life.

Take a moment to think about your experience with the strategy this past week. Is it something you'd like to try again?

- ○ I'll give it a try again
- ○ I'd like to try something new

So while ${e://Field/Heuristic} wasn't very helpful in getting you to exercise more, it was enjoyable and a good fit into your life.

Take a moment to think about your experience with the strategy this past week. Is it something you'd like to try again?

- I'll give it a try again
- I'd like to try something new

${e://Field/Heuristic} was something you didn't really enjoy nor was it a good fit into your life. However, it was helpful in getting you to exercise more.

Take a moment to think about your experience with the strategy this past week. Is it something you'd like to try again?

- I'll give it a try again
- I'd like to try something new

Interesting! It sounds like you didn't really enjoy doing ${e://Field/Heuristic} nor was it helpful in getting you to exercise more, but it fit into your life nicely.

Take a moment to think about your experience with the strategy this past week. Is it something you'd like to try again?

- I'll give it a try again
- I'd like to try something new

Uh ho. It looks like ${e://Field/Heuristic} wasn't enjoyable, helpful, or a good fit into your life.

Take a moment to think about your experience with the strategy this past week. Is it something you'd like to try again to see if it's a strategy that can become useful, or do you feel like you this experience makes you want to try something new?

- I'll give it a try again
- I'd like to try something new

Yay! It looks like ${e://Field/Heuristic} was enjoyable, helpful in getting you to exercise more this week, AND a good fit into your life.

Take a moment to think about your experience with the strategy this past week. Is it something you'd like to try again to nail it down as a long-lasting strategy, or do you feel like you have the hang of it and want to try something new?

- I'll give it a try again
- I'd like to try something new

Well, it looks like ${e://Field/Heuristic} was just so-so at helping you fit exercise into your life.

Take a moment to think about your experience with the strategy this past week. Is it something you'd like to try again to see if it's a strategy that can become useful, or do you feel like you this experience makes you want to try something new?

- I'll give it a try again
- I'd like to try something new

Nice. It takes time for a strategy to become a fixed part of your life and routine. If you're curious to try something else this week, feel free to return to this tool to try out another strategy or module.

Sweet. It's great that you've gotten the hang of this strategy and can continue to turn to it going forward as you continue to work on establishing an exercise routine.

For next week, would you like to try another strategy in this module or another entirely different module?

Stick with this module and explore other

- Strategies to help you fit exercise into your life

Or try something new?

- Strategies to help increase your enjoyment in exercising
- Strategies to reduce negative feelings through exercise
- Strategies to reduce your sense of discomfort while exercising

OK - It seems like ${e://Field/Heuristic} wasn't the best fit - which isn't bad news at all! This process takes time. Now you know what might not work best for you and gives you a chance to find what DOES work.

Would you like to try another strategy in this module or another entirely different module?

Stick with this module and explore other

- Strategies to help you fit exercise into your life

Or try something new?

- Strategies to help increase your enjoyment in exercising
- Strategies to reduce negative feelings through exercise
- Strategies to reduce your sense of discomfort while exercising

That's OK! What is the main reason you did not give the strategy a try?

- I forgot that I selected it
- I was no longer interested in the strategy
- I tried another strategy
- I exercised without using this or any other strategy
- Lack of time to exercise
- Lack of motivation to exercise

This is good to recognize as it can help you to approach next week in a more productive way.

Would you like to try ${e://Field/Heuristic} again and work on setting aside time in your schedule to plan out your strategy and when you'll exercise?

- ○ I'll give it a try again
- ○ I'd like to try something new

This is good to recognize as it can help you to approach this next week in a more productive way.

Would you like to try ${e://Field/Heuristic} again and work on remembering the strategy by writing it down somewhere noticeable or setting a reminder/alarm for yourself on your phone?

- ○ I'll give it a try again
- ○ I'd like to try something new

Great! And keep in mind... If you find that this strategy isn't helpful, that's OK. You're learning what does and doesn't work for you. You can always return to this tool to try out another strategy or module!

Ok! Would you like to try another strategy in this module or another entirely different module?

Stick with this module and explore other

- ○ Strategies to help you fit exercise into your life

Or try something new?

- ○ Strategies to help increase your enjoyment in exercising
- ○ Strategies to reduce negative feelings through exercise
- ○ Strategies to reduce your sense of discomfort while exercising

It seems like ${e://Field/Heuristic} wasn't a good fit - which isn't bad news at all! This process takes time. Now you know what doesn't work for you and gives you a chance to find what DOES work.

Would you like to try another strategy in this module or another entirely different module?

Stick with this module and explore other

- Strategies to help you fit exercise into your life

Or try something new?

- Strategies to help increase your enjoyment in exercising
- Strategies to reduce negative feelings through exercise
- Strategies to reduce your sense of discomfort while exercising

That's great! You were able to exercise regardless of trying out this experiment. Using ${e://Field/Heuristic} to help fit exercise in your life may not be a good strategy for you OR it could be if you had the opportunity to try it out.

Take a moment to think about your experience with exercising this week. Is this experiment something you'd like to try again, as part of your routine, or do you feel like you want to try something new?

Stick with this module and explore other

- Strategies to help you fit exercise into your life

Or try something new?

- Strategies to help increase your enjoyment in exercising
- Strategies to reduce negative feelings through exercise
- Strategies to reduce your sense of discomfort while exercising

THINGS TO DO TO INCREASE ENJOYMENT CHECK IN

Last week you aimed to try to increase your enjoyment of exercise by **${e://Field/Enjoyment}** each time you exercised.

Did you end up giving it a try?

- Yes
- No

Great to hear you gave it a shot!

	Not at all	Not so much	Kind of	A good amount	A lot
How did you like it?	○	○	○	○	○
How helpful was it making exercise a more positive experience for you?	○	○	○	○	○
Did it fit into your life easily?	○	○	○	○	○

So it looks like although you didn't like ${e://Field/Enjoyment} while you exercised all that much, it fit easily into your life and helped you to find exercise more enjoyable. This is important to note, as it can help you to decide what you want to do in the weeks ahead.

Take a moment to think about your experience with this experiment this past week. Is it something you'd like to try again?

- ○ I'll give it a try again
- ○ I'd like to try something new

Good to know! It sounds liked ${e://Field/Enjoyment} while you exercised, but it wasn't a good fit, nor did it help you to find exercise more enjoyable.

Take a moment to think about your experience with this experiment this past week. Is it something you'd like to try again?

- ○ I'll give it a try again
- ○ I'd like to try something new

OK! So ${e://Field/Enjoyment} while you exercised was enjoyable and helped you to find exercise a more positive experience, but it really wasn't a good fit into your life.

Take a moment to think about your experience with this experiment this past week. Is it something you'd like to try again?

- I'll give it a try again
- I'd like to try something new

So while ${e://Field/Enjoyment} while you exercised didn't really make exercise a more positive experience, it was a good fit for your life and something you liked to do.

Take a moment to think about your experience with this experiment this past week. Is it something you'd like to try again?

- I'll give it a try again
- I'd like to try something new

${e://Field/Enjoyment} while you exercised wasn't something you liked nor was it a good fit into your life. However, it was helpful in making exercise an overall more positive experience.

Take a moment to think about your experience with this experiment this past week. Is it something you'd like to try again?

- I'll give it a try again
- I'd like to try something new

Interesting! It sounds like you didn't really like ${e://Field/Enjoyment} while you exercised nor was it helpful in making exercise more enjoyable, but it fit into your life nicely.

Take a moment to think about your experience with this experiment this past week. Is it something you'd like to try again?

- I'll give it a try again
- I'd like to try something new

Well, it looks like ${e://Field/Enjoyment} was just so-so at helping you increase your enjoyment while exercising.

Take a moment to think about your experience with the strategy this past week. Is it something you'd like to try again to see if it's a strategy that can become useful, or do you feel like you this experience makes you want to try something new?

- ◯ I'll give it a try again
- ◯ I'd like to try something new

Uh ho. It looks like ${e://Field/Enjoyment} while you exercised wasn't enjoyable, helpful to making exercise a more positive experience, or a good fit into your life.

Take a moment to think about your experience with this experiment this past week. Is it something you'd like to try again to see if it's something that can become useful, or do you feel like you this experience makes you want to try something new?

- ◯ I'll give it a try again
- ◯ I'd like to try something new

Yay! It looks like ${e://Field/Enjoyment} while you exercise was enjoyable, helpful in making exercise a more positive experience, AND a good fit into your life.

Take a moment to think about your experience with this experiment this past week. Is it something you'd like to try again to nail it down as a long-lasting habit, or do you feel like you have the hang of it and want to try something new?

- ◯ I'll give it a try again
- ◯ I'd like to try something new

Nice. It takes time for something to become a fixed part of your life and routine. If you're curious to try something else this week, feel free to return to this tool to try out another experiment to make exercise more enjoyable or an entirely different module.

Sweet. It's great that you've gotten the hang of this practice and can continue to turn to it going forward to make exercise a positive experience. Because, ultimately, if you like something you're more likely to stick to it!

For next week, would you like to try another practice in this module to find other ways to make exercise a positive experience or select another entirely different module?

Stick with this module and explore other

- ○ Strategies to help increase your enjoyment in exercising

Or try something new?

- ○ Strategies to help you fit exercise into your life
- ○ Strategies to reduce negative feelings through exercise
- ○ Strategies to reduce your sense of discomfort while exercising

OK - It seems like ${e://Field/Enjoyment} wasn't the best fit - which isn't bad news at all! This process takes time. Now you know what might not work best for you and gives you a chance to find what DOES work.

For next week, would you like to try another practice in this module to find other ways to make exercise a positive experience or select another entirely different module?

Stick with this module and explore other

- ○ Strategies to help increase your enjoyment in exercising

Or try something new?

- ○ Strategies to help you fit exercise into your life
- ○ Strategies to reduce negative feelings through exercise
- ○ Strategies to reduce your sense of discomfort while exercising

That's OK! What is the main reason you did not ${e://Field/Enjoyment} while you exercised this week?

- ○ I forgot that I selected it
- ○ I was no longer interested in the strategy

- I tried another strategy
- I exercised without using this or any other strategy
- Lack of time to exercise
- Lack of motivation to exercise

Cool to see that you gave exercise a go even without trying out this experiment. ${e://Field/Enjoyment} while you exercise may not be a good fit (which is A-OK as it takes time to find out ways to make exercise enjoyable) OR it could still be a good fit if you gave it a chance.

Take a moment to think about your experience with exercising this week. Is this experiment something you'd like to try again, as part of your routine, or do you feel like you want to try something new?

- I'll give it a try again
- I'd like to try something new

This is good to recognize as it can help you to approach this next week in a more productive way.

Would you like to try ${e://Field/Enjoyment} while you exercise again and work on setting aside time in your schedule to plan out when you'll exercise and how to fit in the experiment when you do?

- I'll give it a try again
- I'd like to try something new

This is good to recognize as it can help you to approach this next week in a more productive way.

Would you like to try ${e://Field/Enjoyment} while you exercise again and work on remembering the experiment by writing it down somewhere noticeable or setting a reminder/alarm for yourself on your phone?

- I'll give it a try again
- I'd like to try something new

This is good to recognize as it can help you to approach this next week in a more productive way.

Would you like to try ${e://Field/Enjoyment} while you exercise again and work on starting to exercise for just a few minutes (even if you're not feeling entirely motivated) and see if what you selected gives you the boost to keep going?

- I'll give it a try again
- I'd like to try something new

Great! And keep in mind... If you find that this strategy isn't helpful, that's OK. You're learning what does and doesn't work for you. You can always return to this tool to try out another strategy or module!

Cool! Would you like to try another strategy in this module or another entirely different module?

Stick with this module and explore other

- Strategies to help increase your enjoyment in exercising

Or try something new?

- Strategies to help you fit exercise into your life
- Strategies to reduce negative feelings through exercise
- Strategies to reduce your sense of discomfort while exercising

It seems like ${e://Field/Enjoyment} while you exercise isn't a good fit - which isn't bad news at all! It takes time to find out ways to make exercise enjoyable. Now you know what doesn't work for you and gives you a chance to find what DOES work.

Would you like to try another experiment in this module to see if there are other ways to make exercise enjoyable or do you want to go for another entirely different module?

Stick with this module and explore other

- ○ Strategies to help increase your enjoyment in exercising

Or try something new?

- ○ Strategies to help you fit exercise into your life
- ○ Strategies to reduce negative feelings through exercise
- ○ Strategies to reduce your sense of discomfort while exercising

NEGATIVE FEELINGS CHECK IN

Over this past week you attempted to and see if exercise impacted **${e://Field/Negative}**, particularly by the time you finished exercising.

Did you end up giving it a try?

- ○ Yes
- ○ No

Cool!

	Not at all	Not so much	Kind of	A good amount	A lot
How did you like focusing on how exercise impacted ${e://Field/Negative}?	○	○	○	○	○
How helpful was exercise at improving ${e://Field/Negative}?	○	○	○	○	○
Did taking the time to think about exercise's impact on ${e://Field/Negative} fit into your life easily?	○	○	○	○	○

On one hand you didn't like focusing on how exercise impacted ${e://Field/Negative}, but on the other taking the time to do so fit easily into your life and you found that exercise improved this negative feeling. This is important to note, as it can help you to decide what you want to do in the weeks ahead.

Take a moment to think about your experience with this experiment this past week. Is it something you'd like to try again?

- I'll give it a try again
- I'd like to try something new

Noted! You liked focusing on exercise's impact on ${e://Field/Negative}, but taking the time to do it didn't fit into your life nor did you find that exercise improved this negative feeling.

Take a moment to think about your experience with this experiment this past week. Is it something you'd like to try again?

- I'll give it a try again
- I'd like to try something new

Good to know! So focusing on exercise's impact on ${e://Field/Negative} was enjoyable and you found that it improved with exercise, but taking the time to do so didn't fit well into your life.

Take a moment to think about your experience with this experiment this past week. Is it something you'd like to try again?

- I'll give it a try again
- I'd like to try something new

OK, so while you didn't find that exercise improved ${e://Field/Negative}, taking the time to think about it fit into your life and it was something you liked to do.

Take a moment to think about your experience with this experiment this past week, keeping in mind that exercise's impact on negative feelings can change over time. Is it something you'd like to try again?

- I'll give it a try again
- I'd like to try something new

So there were some pros and cons... Taking time to think about exercise's impact on ${e://Field/Negative} wasn't something you liked to do nor did it fit into your life. However, you did learn that exercise improved this negative experience.

Take a moment to think about your experience with this experiment this past week. Is it something you'd like to try again?

- I'll give it a try again
- I'd like to try something new

This is good feedback. It sounds like you didn't really like to take time to think about how exercise impacts ${e://Field/Negative} nor did you find that exercise improved this negative experience, but the practice itself fit into your life nicely.

Take a moment to think about your experience with this experiment this past week, keeping in mind that exercise's impact on negative feelings can change over time. Is it something you'd like to try again?

- I'll give it a try again
- I'd like to try something new

Bummer. It looks like thinking about how exercise impacts ${e://Field/Negative} wasn't enjoyable or a good fit into your life, nor did you find that exercise improved this negative feeling.

Take a moment to think about your experience with this experiment this past week. Is it something you'd like to try again, to see if it's something that can become useful, or do

you feel like you this experience makes you want to try something new?

- I'll give it a try again
- I'd like to try something new

Right on! It looks like thinking about exercise's impact on ${e://Field/Negative} was enjoyable and a good fit into your life, plus you found that exercise improved this negative feelings.

Take a moment to think about your experience with this experiment this past week. Is it something you'd like to try again, to nail it down as a long-lasting practice, or do you feel like you have the hang of it and want to try something new?

- I'll give it a try again
- I'd like to try something new

Well, it looks like ${e://Field/Negative} was just so-so at helping you reduce negative feelings through exercise.

Take a moment to think about your experience with the strategy this past week. Is it something you'd like to try again to see if it's a strategy that can become useful, or do you feel like you this experience makes you want to try something new?

- I'll give it a try again
- I'd like to try something new

Nice. It takes time for this kind of practice to become a fixed part of your life and routine. If you're curious to try something else this week, feel free to return to this tool to try and see exercise's impact on another negative feeling or an entirely different module.

Nice! It's great that you've gotten the hang of this experiment and can continue to turn to it going forward to remind yourself of how exercise can improve negative feelings like ${e://Field/Negative}.

For next week, would you like to try and see how exercise impacts other negative feelings found in this module or select another entirely different module?

Stick with this module and explore other

- ○ Strategies to reduce negative feelings through exercise

Or try something new?

- ○ Strategies to reduce your sense of discomfort while exercising
- ○ Strategies to help you fit exercise into your life
- ○ Strategies to help increase your enjoyment in exercising

OK - It seems like ${e://Field/Negative} wasn't the best fit - which isn't bad news at all! This process takes time. Now you know what might not work best for you and gives you a chance to find what DOES work.

For next week, would you like to try and see how exercise impacts other negative feelings found in this module or select another entirely different module?

Stick with this module and explore other

- ○ Strategies to reduce negative feelings through exercise

Or try something new?

- ○ Strategies to reduce your sense of discomfort while exercising
- ○ Strategies to help you fit exercise into your life
- ○ Strategies to help increase your enjoyment in exercising

No worries! What is the main reason you did not take time to think about how exercise impacts ${e://Field/Negative} this week?

- ○ I forgot that I selected it
- ○ I was no longer interested in the strategy
- ○ I tried another strategy
- ○ I exercised without using this or any other strategy
- ○ Lack of time to exercise

○ Lack of motivation to exercise

Congrats! You were able to exercise regardless of trying out this experiment. Thinking through how exercise impacts ${e://Field/Negative} may not be a good fit (totally cool!) OR it could still be a good fit if you had the opportunity to try it out.

Take a moment to think about your experience with exercising this week. Is thinking through how exercise impacts ${e://Field/Negative} something you'd like to try again, as part of your routine, or do you feel like you want to try something new?

○ I'll give it a try again
○ I'd like to try something new

That's understandable! It's good to recognize this as a reason you didn't explore exercise's impact on ${e://Field/Negative} as it can help you to approach this next week in a more productive way.

Would you like to try to this strategy again by working on setting aside time in your schedule to exercise and fit in the experiment when you do?

○ I'll give it a try again
○ I'd like to try something new

That's understandable! It's good to recognize this as a reason you didn't explore exercise's impact on ${e://Field/Negative} as it can help you to approach this next week in a more productive way.

Would you like to try to think about this relationship again and work remembering the experiment by writing it down somewhere noticeable or setting a reminder/alarm for yourself on your phone?

○ I'll give it a try again
○ I'd like to try something new

That's understandable! It's good to recognize this as a reason you didn't explore exercise's impact on ${e://Field/Negative} as it can help you to approach this next week in a more productive way.

Would you like to try to think about this relationship again and work on starting to exercise for just a few minutes (even if you're not feeling entirely motivated) and see if this negative feeling improves?

- I'll give it a try again
- I'd like to try something new

Great! And keep in mind... If you find that thinking through this isn't helpful, that's OK. You're learning what does and doesn't work for you. You can always return to this tool to see exercise's impact on another negative feeling or a completely different module entirely!

Cool! Would you like to explore how exercise impacts another negative feeling in this module or try out another entirely different strategy?

Stick with this module and explore other

- Strategies to reduce negative feelings through exercise

Or try something new?

- Strategies to help you fit exercise into your life
- Strategies to help increase your enjoyment in exercising
- Strategies to reduce your sense of discomfort while exercising

Hey, you learned something! It seems like thinking about how exercise impacts ${e://Field/Negative} isn't a good fit. It takes time to find out what is a helpful tool in establishing a regular exercise routine. So now that you know this isn't right for you, you now can find out what DOES.

Would you like to choose another negative feeling in this module to see how exercise might improve it or do you want to go for another entirely different module?

Stick with this module and explore other

- ○ Strategies to reduce negative feelings through exercise

Or try something new?

- ○ Strategies to help you fit exercise into your life
- ○ Strategies to help increase your enjoyment in exercising
- ○ Strategies to reduce your sense of discomfort while exercising

REFRAME DISCOMFORT CHECK IN

Over this past week you set an intention to reframe your experience of **${e://Field/Discomfort}** during exercise in a positive or neutral way in order to reduce discomfort.

Did you try it out?

- ○ Yes
- ○ No

Right on!

	Not at all	Not so much	Kind of	A good amount	A lot
How did you like thinking of your experience of ${e://Field/Discomfort} during exercise in a positive/neutral way?	○	○	○	○	○
How helpful was reframing ${e://Field/Discomfort} at reducing feelings of discomfort while you exercised?	○	○	○	○	○

	Not at all	Not so much	Kind of	A good amount	A lot
Did taking the time to reframe your experience of ${e://Field/Discomfort} during exercise fit into your life easily?	◯	◯	◯	◯	◯

Good to know. It sounds like you didn't like reframing your experience of ${e://Field/Discomfort} during exercise, but found that taking the time to do so fit easily into your life and you found that it reduced the discomfort you felt during exercise. This is important to note, as it can help you to decide what you want to do in the weeks ahead.

Take a moment to think about your experience with this experiment this past week. Is it something you'd like to try again?

◯ I'll give it a try again

◯ I'd like to try something new

Helpful feedback! You enjoyed reframing your experience of ${e://Field/Discomfort} during exercise, but taking the time to do so didn't fit into your life nor did you find that it reduced the discomfort you felt during exercise.

Take a moment to think about your experience with this experiment this past week. Is it something you'd like to try again?

◯ I'll give it a try again

◯ I'd like to try something new

Looks like there were some pros and cons. Reframing your experience of ${e://Field/Discomfort} during exercise was enjoyable and you found that it reduced feelings of discomfort, but taking the time to do so didn't fit well into your life.

Take a moment to think about your experience with this experiment this past week. Is it something you'd like to try again?

- I'll give it a try again
- I'd like to try something new

Interesting! While you didn't find that your feelings of discomfort reduced during exercise when you reframed your experience of ${e://Field/Discomfort}, taking the time to think about it in a more positive/neutral way fit into your life and it was something you liked to do.

Take a moment to think about your experience with this experiment this past week, keeping in mind that feelings of discomfort during exercise can change over time. Is it something you'd like to try again?

- I'll give it a try again
- I'd like to try something new

Got it. So, taking time to think reframe your experience of ${e://Field/Discomfort} during exercise wasn't something you liked to do nor did it fit into your life. However, you did find that your feelings of discomfort during exercise improved.

Take a moment to think about your experience with this experiment this past week. Is it something you'd like to try again?

- I'll give it a try again
- I'd like to try something new

It sounds like you didn't really like to take time to reframe your experience of ${e://Field/Discomfort} during exercise nor did you find that it reduced feelings of discomfort. However, the practice itself fit into your life nicely.

Take a moment to think about your experience with this experiment this past week, keeping in mind that feelings of discomfort during exercise can change over time. Is it something you'd like to try again?

- I'll give it a try again

- I'd like to try something new

Even negative feedback is good feedback! You're learning what doesn't work which is useful. To summarize, it looks like reframing your experience of ${e://Field/Discomfort} during exercise wasn't enjoyable or a good fit into your life, nor did you find that feelings of discomfort during exercise improved when doing so.

Take a moment to think about your experience with this experiment this past week. Is it something you'd like to try again, to see if it's something that can become useful, or do you feel like you this experience makes you want to try something new?

- I'll give it a try again
- I'd like to try something new

Awesome! It looks like reframing your experience of ${e://Field/Discomfort} during exercise was enjoyable and a good fit into your life, plus you found that it helped to reduce feelings of discomfort.

Take a moment to think about your experience with this experiment this past week. Is it something you'd like to try again, to nail it down as a long-lasting practice, or do you feel like you have the hang of it and want to try something new?

- I'll give it a try again
- I'd like to try something new

Well, it looks like reframing ${e://Field/Discomfort} was just so-so at reducing your sense of discomfort while exercising.

Take a moment to think about your experience with this experiment this past week. Is it something you'd like to try again, to nail it down as a long-lasting practice, or do you feel like you have the hang of it and want to try something new?

- I'll give it a try again
- I'd like to try something new

Nice. It takes time for this kind of practice to become a fixed part of your life and routine. If you're curious to try something else this week, feel free to return to this tool to try and reframe another discomfort listed or to give an entirely different module a go.

It's great that you've gotten the hang of this experiment and can continue to turn to it going forward to reframe your experience of ${e://Field/Discomfort} during exercise in a way that can reduce discomfort.

For next week, would you like to try and reframe another experience of discomfort during exercise from this module or select another entirely different module?

Stick with this module and explore other

- ◯ Strategies to reduce your sense of discomfort while exercising

Or try something new?

- ◯ Strategies to help you fit exercise into your life
- ◯ Strategies to help increase your enjoyment in exercising
- ◯ Strategies to reduce negative feelings through exercise

OK - It seems like ${e://Field/Discomfort} wasn't the best fit - which isn't bad news at all! This process takes time. Now you know what might not work best for you and gives you a chance to find what DOES work.

For next week, would you like to try and reframe another experience of discomfort during exercise from this module or select another entirely different module?

Stick with this module and explore other

- ◯ Strategies to reduce your sense of discomfort while exercising

Or try something new?

- ◯ Strategies to help you fit exercise into your life
- ◯ Strategies to help increase your enjoyment in exercising
- ◯ Strategies to reduce negative feelings through exercise

No worries! What is the main reason you did not take time to reframe your experience of ${e://Field/Discomfort} during exercise this week?

- I forgot that I selected it
- I was no longer interested in the strategy
- I tried another strategy
- I exercised without using this or any other strategy
- Lack of time to exercise
- Lack of motivation to exercise

That's great! You were able to exercise regardless of trying out this experiment. Reframing your experience of ${e://Field/Discomfort} during exercise may not be a good fit OR it could be if you had the opportunity to try it out.

Take a moment to think about your experience with exercising this week. Is reframing this discomfort during exercise something you'd like to try again, as part of your routine, or do you feel like you want to try something new?

- I'll give it a try again
- I'd like to try something new

It happens! It's good to be aware that this as a reason you didn't reframe your experience of ${e://Field/Discomfort} during exercise as it can help you to approach this next week in a more productive way.

Would you like to try to think about this relationship again and work on setting aside time in your schedule to plan out when you'll exercise and how to fit in the experiment when you do?

- I'll give it a try again
- I'd like to try something new

It happens! It's good to be aware that this as a reason you didn't reframe your experience of ${e://Field/Discomfort} during exercise as it can help you to approach this next week in a more productive way.

Would you like to try to think about this relationship again and work on remembering the experiment by writing it down somewhere noticeable or setting a reminder/alarm for yourself on your phone ?

- I'll give it a try again
- I'd like to try something new

It happens! It's good to be aware that this as a reason you didn't reframe your experience of ${e://Field/Discomfort} during exercise as it can help you to approach this next week in a more productive way.

Would you like to try to think about this relationship again and work on starting to exercise for just a few minutes (even if you're not feeling entirely motivated) and see if reframing this discomfort boosts your motivation?

- I'll give it a try again
- I'd like to try something new

Great! Taking more time with an experiment can definitely help you to make it a more concrete part of your life and/or continue to see if it's a good fit and helpful for you. And keep in mind... If you find that reframing ${e://Field/Discomfort} during exercise isn't helpful, that's ok. You can always return to this tool to try and reframe another discomfort listed or a completely different module entirely!

That works! Would you like to reframe another discomfort during exercise from this module or try out another entirely different module?

Stick with this module and explore other

- Strategies to reduce your sense of discomfort while exercising

Or try something new?

- Strategies to help you fit exercise into your life
- Strategies to help increase your enjoyment in exercising
- Strategies to reduce negative feelings through exercise

Discovering what you're not interested in is useful information! You're one step closer to finding what WILL work for you which takes time.

So now that you know that reframing ${e://Field/Discomfort} during exercise isn't right for you, would you like to choose another discomfort from this module to see if it improves your feelings of discomfort during exercise or do you want to go for another entirely different module?

Stick with this module and explore other

- Strategies to reduce your sense of discomfort while exercising

Or try something new?

- Strategies to help you fit exercise into your life
- Strategies to help increase your enjoyment in exercising
- Strategies to reduce negative feelings through exercise

DEVELOPING PLANS MODULES_1

Alright! So, you want to experiment with strategies to help you fit exercise into your life. We've got a ton of them for you to try.

Hover over each option and read through what each entails. Then, **pick one** that sounds the most appealing, helpful, and/or interesting to you. (Don't worry, you'll have plenty of chances to try out more in the future!)

Keep in mind that it's OK if you select one that sounds good now but, after you try it, it doesn't work for you. You are learning, which is what matters most. You are always able to come back and try something else!

Hover over the text to see a description of the practice.

- Prep the night before
- Have a "plan c"
- Start with 10
- Make a date with exercise
- Seize the morning
- A sweat a day
- Workout with a buddy
- Reward yourself!
- Create visual reminders
- Stick to a 2-day rule
- Never miss a Monday
- Exercise in short bursts
- Exercise shoe trick
- Be a weekend warrior

DEVELOPING PLANS MODULES_2

Cool! You selected

${e://Field/Heuristic}

to experiment with and try this week. The goal of this is to help you to fit in your exercise selections in your life and, ultimately, reach your weekly MVPA goals.

Specifically, this strategy asks that you **${e://Field/Heuristic_description}**

If the strategy you selected doesn't end up working for you, that's OK! Not every strategy will work for everyone and it can take some trial-and-error to find what works for you and your unique circumstances. Next time you check in with **Reflect** you can select something else.

Your plan this week is to experiment with strategies to increase your enjoyment in exercising. Great choice! Because if you learn to love exercise, you'll feel more driven to do it, particularly even if you feel tired or are not in the mood.

Start by reviewing the following positive experiences people often link with exercise. Hover over each one for a more detailed description. Which ones would also be a positive experience for you?

Next, **pick one** you can experiment with while exercising this week. If there is something you would want to try but it's not listed, select "Other" and specify.

Hover over the text to see a description of the practice.

- ○ Listening to a podcast/audiobook
- ○ Listening to music
- ○ Watching TV
- ○ Enjoying the outdoors
- ○ Enjoying the weather
- ○ Getting some alone time
- ○ Socializing with others
- ○ Enjoying an active hobby
- ○ Being able to decompress/Unwind
- ○ [ ] Other

DEVELOPING PLANS MODULES_3

Nice! You're going to try to
${e://Field/Enjoyment}
when you exercise this week. ${e://Field/Enjoyment_description}.

While experimenting, notice if what you selected makes exercise more enjoyable. If it does, wonderful! You're a step closer in making exercise a positive experience. If it doesn't, no problem! You learned something and are free to find what does work for you. And, remember, you are able to return to **Reflect** and explore alternatives.

Nice choice! You are going to try out strategies to reduce negative feelings through exercise. Exercise impacts both physical and mental health. This strategy is a good way to experiment with how negative feelings you may experience change with exercise and if that ultimately encourages you to move more.

Review each of the negative feelings below and think about which ones may be impacted by exercise for you. Then, **select one** that resonates most. If there is something you'd like to add, select "Other" and specify.

- feeling anxious
- feeling stressed
- feeling sad/depressed
- feeling fatigued
- feeling discouraged
- feeling like you're not living up to identity as an exerciser
- feeling physical aches and pains
- feelings associated with a particular illness or condition you have (Type the illness/condition below) ______
- ______ Other negative feelings

Alright! So, this week you are going to focus on your experience with **${q://QID15/ChoiceGroup/SelectedChoicesTextEntry}**, before, during, and after you exercise. Think about how what you selected changes (or doesn't) over time - there is no "right" experience with this.

If you find that there is an improvement, fantastic! Exercise could be seen as a healthy way to cope with this negative feeling. If you find no change or that your feelings worsen, it's OK. You've discovered something about yourself. You can come back to **Reflect** and experiment with another strategy that will be more helpful.

OK, so you are wanting to experiment with strategies to reduce experiences of discomfort while exercising.

There are a lot of experiences that come up during exercise that can be seen as negative. Look over what we've listed below and think about which you find to be uncomfortable, unpleasant, discouraging, etc. when you exercise. Next, **select one** that stands out to you the most.

- ○ Soreness 😫
- ○ Fatigue 😴
- ○ Feeling hot 🥵
- ○ Feeling sweaty 😰
- ○ Not being able to perform well 😔
- ○ Apathy 🥱

DEVELOPING PLANS MODULES_4

OK, so **${e://Field/Discomfort}** is something that comes up for you as a negative experience when you exercise. Changing how we think about these kinds of experiences can help to reduce barriers to exercise and encourage us to exercise regularly.

This week, when you experience ${e://Field/Discomfort} during exercise, we want you to try and reframe it as a neutral or even positive experience. For example, for ${e://Field/Discomfort} you could reframe it as ${e://Field/Discomfort_reframe}.

If you find that rethinking this experience in a neutral or positive way works for you, amazing! If you find that it doesn't, give it a few chances and always know you can come back to **Reflect** and try another strategy that may be more helpful for you.

Summary Module

Let's recap! To reach your MVPA goal this week you are:

- Aiming to get your active minutes through **${e://Field/MVPA_ratio}**.
- Focusing on trying **${e://Field/Exercise_all}** as often as you can this week for aerobic exercise.
- Experimenting with **${e://Field/Strategy}** as a way to help you to exercise regularly

We'll check in with you each time you exercise this week via your Fitbit to see how your experiment is going. So when you see a message asking you about **Reflect** we're seeing if **${e://Field/Summary_description}**. Check your watch and Fitbit app daily to see how your active minutes stack up and how the moderate-vigorous intensity ratio and the exercises you selected are working for you.

Remember to take the pressure out of it! Much like prior weeks, see this week as another chance to learn what might be helpful for you as you continue to establish an exercise routine. Jot down your experiences - good and bad - in a place that is convenient and easy to remember, like your planner or on your phone. This way you can refer to them during next week's check-in.

UCSD IRB# 200733

Powered by Qualtrics
