# Supplementary material for: Optimizing and Testing an Individualized and Adaptive Physical Activity Digital Health Intervention: Protocol for a Control Optimization Trial Embedded Within a Randomized Controlled Trial
Source: JMIR Res Protoc. 2025 Aug 15;14:e70599. doi: 10.2196/70599 (PMC12397713; doi:10.2196/70599)
Supplement: Multimedia Appendix 4 [file resprot_v14i1e70599_app4.pdf]

YourMove Reflect Summary for the Week of [date]

From YourMove Reflect Summary
Date
To

Thanks for completing this week's **Reflect!** Here is your summary for the week of [date]:

Let's recap! To reach your MVPA goal this week you are:

- Aiming to get your active minutes through **mostly moderate-intensity activity only.**
- Focusing on trying **walking, elliptical, and swimming** as often as you can this week for aerobic exercise.
- Experimenting with **strategies to help you fit exercise into your life** as a way to help you to exercise regularly

We'll check in with you each time you exercise this week via your Fitbit to see how your experiment is going. So when you see a message asking you about **Reflect** we're seeing if **you tried out stick to a 2-day rule and, if so, was it helpful in getting you to exercise?.**

Check your watch and Fitbit app daily to see how your active minutes stack up and how the moderate-vigorous intensity ratio and the exercises you selected are working for you.

Remember to take the pressure out of it! Much like prior weeks, see this week as another chance to learn what might be helpful for you as you continue to establish an exercise routine. Jot down your experiences - good and bad - in a place that is convenient and easy to remember, like your planner or on your phone. This way you can refer to them during next week's check-in.

All the best,
YourMove Study Staff
