# Supplementary material for: Optimizing and Testing an Individualized and Adaptive Physical Activity Digital Health Intervention: Protocol for a Control Optimization Trial Embedded Within a Randomized Controlled Trial
Source: JMIR Res Protoc. 2025 Aug 15;14:e70599. doi: 10.2196/70599 (PMC12397713; doi:10.2196/70599)
Supplement: Multimedia Appendix 5 [file resprot_v14i1e70599_app5.pdf]

Strategies to help you fit exercise into your life	
**Heuristic**	**Description**
prep the night before	lay out everything you need for your planned workout for the next day (e.g., clothes, shoes, water bottle) and put it in a place you'll see it in the morning.
have a "plan c"	take a minute to jot down your "plan A" to exercise this week, your "plan B" if that doesn't pan out, AND a "plan C" if both aren't possible. This way, each time you have a plan to exercise you can do so, regardless of what life throws your way.
start with 10	set a timer for 10 minutes and start your workout until at least time runs out. If you're still feeling unmotivated to continue when the timer goes off, give yourself permission to stop. However, odds are, you'll be motivated to finish your workout!
make a date with exercise	add when you plan to exercise to the same place you keep track of your meetings, appointments, and other commitments so that it's just as much a priority.
seize the morning	set your alarm clock to 30-60 minutes earlier than usual and use the time to workout. If you're an early bird, you'll capitalize on your morning energy. If you're a busy bee, you'll be carving out time you otherwise would be sleeping so that you don't have to add something more to your jam-packed day.
a sweat a day	keep it simple and commit to a sweat-a-day! Whether that be a brisk walk after dinner, getting yard work done on the weekend, or enjoying an active day outside with loved ones, you'll get in active minutes and establish a habit of exercising each day.
workout with a buddy	plan to workout with a friend and/or team up with a friend to hold each other accountable to one another's workout schedule. You'll more likely to stick with and challenge yourself during your workouts.
reward yourself!	pick a reasonable reward and gift it to yourself each time you reach your weekly exercise goal or, better yet, when you reach your goals for an entire month.
create visual reminders	use sticky notes to jot down reminders to exercise, and stick them in places you see every day - the more the better!
stick to a 2-day rule	stay consistent with exercise by making it a rule to never go more than 2 days without exercising.
never miss a Monday	start your week off right by exercising every Monday, no excuses!
exercise in short bursts	divide your workouts into small but meaningful bouts (10+ minutes) to make your weekly exercise goal more manageable.
be a weekend warrior	take advantage of a little extra free time on the weekends and commit to working out every Saturday and Sunday.
exercise shoe trick	put on your exercise shoes around the time of day you plan to exercise (e.g., first thing in the morning, right after work) and don't take them off until your workout is completed.

Strategies to help increase your enjoyment in exercising	
**Strategy**	**Description**
listening to a podcast/audiobook	Specifically, plug in a podcast or audiobook you are currently enjoying to make your workout fly by
listening to music	Specifically, turn up your favorite high-energy music to make your workout more enjoyable and give you and energy boost
watching TV	Specifically, when exercising indoors, turn on your favorite show to keep yourself entertained and help the time fly by
enjoying the outdoors	Specifically, take your workout outside to enjoy a change of scenery
enjoying the weather	Specifically, take your workout outside to enjoy the weather
getting some alone time	Specifically, make working out your "me time"
socializing with others	Specifically, have someone join you while exercising, either in-person or over the phone, to make working out your "social hour"
enjoying an active hobby	Specifically, make working out double as time to enjoy exercise as an active hobby
being able to decompress/unwind	Specifically, make the time you spend exercising double as time to decompress and unwind

Strategies to reduce your sense of discomfort while exercising	
**Discomfort**	**Reframe example**
soreness	trying a new exercise; increasing load; building strength
fatigue	accomplishing a challenging workout
feeling hot	body's natural response to exercise; one way to show you are working hard
feeling sweaty	body's natural way to keep cool; one way to show you are working hard
not being able to perform well	progress not perfection; trying something new; room to grow
apathy	opportunity to find and try a different way to exercise

Strategies to reduce negative feelings through exercise
**Negative Feeling**
feeling anxious
feeling stressed
feeling sad/depressed
feeling fatigued
feeling discouraged
feeling like you're not living up to identity as an exerciser
feeling physical aches and pains
feelings associated with a particular illness or condition you have (w/ text entry)
Other negative feelings (w/ text entry)
