# Supplementary figures and images for: Optimizing and Testing an Individualized and Adaptive Physical Activity Digital Health Intervention: Protocol for a Control Optimization Trial Embedded Within a Randomized Controlled Trial
Source: JMIR Res Protoc. 2025 Aug 15;14:e70599. doi: 10.2196/70599 (PMC12397713; doi:10.2196/70599)

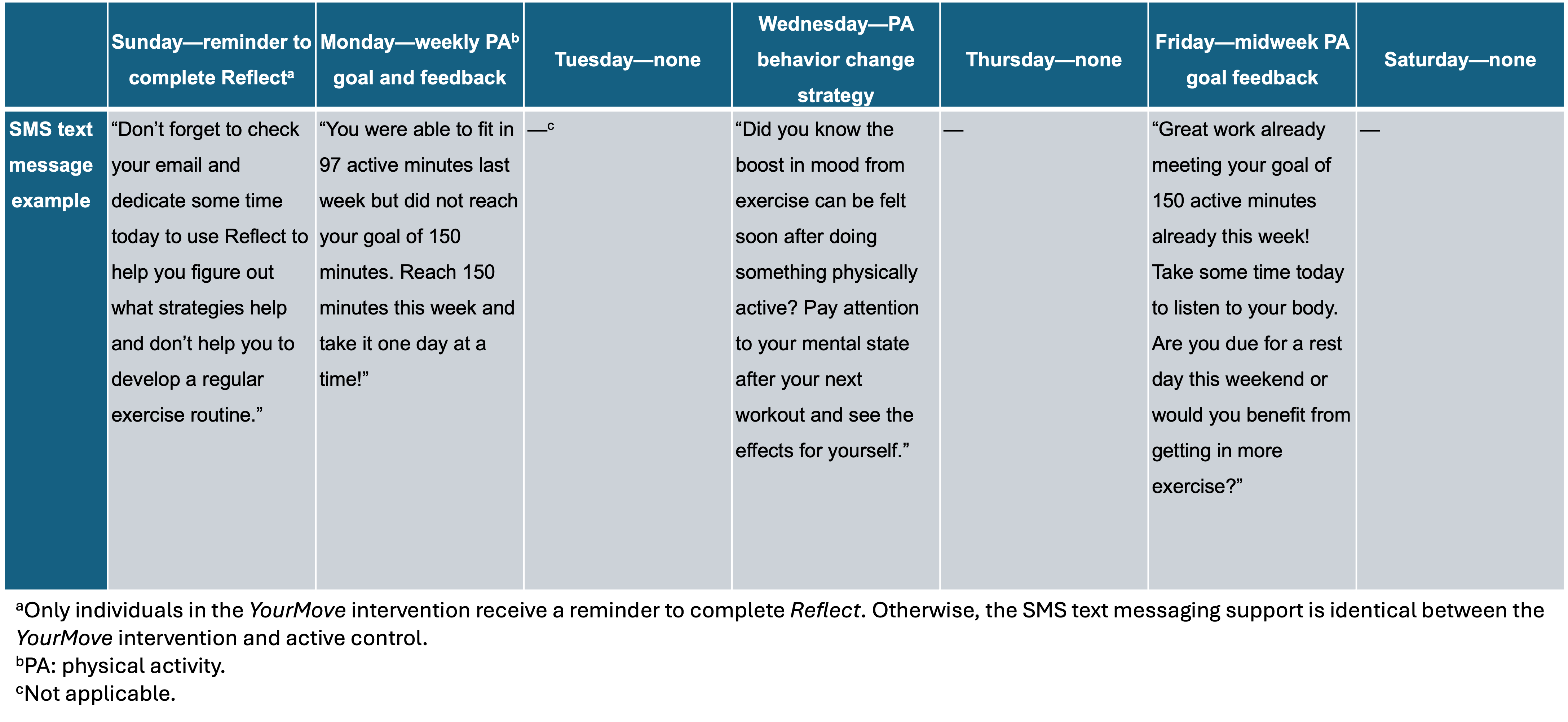

Supplement: Multimedia Appendix 8 [file resprot_v14i1e70599_app8.png]
