# Supplementary material for: Optimizing and Testing an Individualized and Adaptive Physical Activity Digital Health Intervention: Protocol for a Control Optimization Trial Embedded Within a Randomized Controlled Trial
Source: JMIR Res Protoc. 2025 Aug 15;14:e70599. doi: 10.2196/70599 (PMC12397713; doi:10.2196/70599)
Supplement: Multimedia Appendix 9 [file resprot_v14i1e70599_app9.pdf]

SUMMARY STATEMENT

PROGRAM CONTACT:
April Oh
240-276-6709
ohay@mail.nih.gov

(Privileged Communication)

Release Date: **02/11/2020**
Revised Date:

Application Number: **1 R01 CA244777-01A1**

Principal Investigator

HEKLER, ERIC B

Applicant Organization: UNIVERSITY OF CALIFORNIA, SAN DIEGO

Review Group: **PRDP**
Psychosocial Risk and Disease Prevention Study Section

Meeting Date: **01/27/2020**
Council: **MAY 2020**
Requested Start: **07/01/2020**

RFA/PA: **PAR18-559**
PCC: **0OCI**

Project Title: **Optimizing Individualized and Adaptive mHealth Interventions via Control Systems Engineering Methods**
SRG Action: **Impact Score:18 Percentile:7**
Next Steps: **Visit https://grants.nih.gov/grants/next_steps.htm**
Human Subjects: **48-At time of award, restrictions will apply**
Animal Subjects: **10-No live vertebrate animals involved for competing appl.**
Gender: **1A-Both genders, scientifically acceptable**
Minority: **1A-Minorities and non-minorities, scientifically acceptable**
Age: **3A-No children included, scientifically acceptable**

Project Year	Direct Costs Requested	Estimated Total Cost
1	[redacted]	[redacted]
2		
3		
4		
5		
TOTAL	[redacted]	[redacted]

ADMINISTRATIVE BUDGET NOTE: The budget shown is the requested budget and has not been adjusted to reflect any recommendations made by reviewers. If an award is planned, the costs will be calculated by Institute grants management staff based on the recommendations outlined below in the COMMITTEE BUDGET RECOMMENDATIONS section.

1R01CA244777-01A1 Hekler, Eric

PROTECTION OF HUMAN SUBJECTS UNACCEPTABLE

RESUME AND SUMMARY OF DISCUSSION: This application seeks support to prevent cancer by testing an adaptive physical activity walking intervention among inactive overweight and obese adults using a control optimization trial (COT) design. This resubmitted study was highly responsive to previous critiques resulting in work that the review panel opined now offers high impact to significantly addresses the challenging topic of sedentary behavior among overweight and obese adults to ultimately reduce cancer risk. Many strengths were again discussed: the outstanding scientific rigor of the convincing premise of the COT design that holds high promise to identify the effective determinants of the physical activity intervention using a compelling adaptive method and focus on obese adults in work informed by strong pilot work; the exceptional team of investigators, excellent MPI organization including an experienced and impressive PI and systems engineer expertise further bolstered by an exceptional research environment; exceptional innovation inherent in the COT design which perpetually adapts the intervention; and outstanding scientific rigor of the approach strengthened by the test of the unique adaptive intervention, work informed by strong preliminary pilot data, objective physical activity assessment, work well grounded in social cognitive behavioral theory, and excellent usability, acceptability, randomization and dissemination plans. A few minor weaknesses were discussed: lack of justification for the collection of HbA1c, missed opportunity to harness the role of social support, and safety concern regarding the conduct of fitness tests on sedentary overweight/obese adults. A minority opinion urged inclusion of normal weight adults; other reviewers disagreed noting the impact of physical activity interventions on cancer prevention has shown evidence among overweight/obese adults. In sum, the reviewers expressed keen enthusiasm for this high impact work with notable strengths and a few minor weaknesses.

DESCRIPTION (provided by applicant): Strong evidence indicates physical activity (PA) reduces risk of bladder, breast, colon, endometrium, esophagus, gastric, and renal cancer, and there is moderate evidence for lung cancer. Individuals aged 40+ who are inactive are at high risk of developing cancers 58,65 but only 1/3 meet guidelines for PA;5-15 thus, they are an important group to target. While effective PA interventions exist, interventions often work only for some individuals or only for a limited time,16-18 thus establishing the need for interventions that can account for dynamic, idiosyncratic PA determinants in order to support each person's PA. In response, we developed JustWalk, a modular adaptive mobile health (mHealth) intervention that makes daily N-of-1 adjustments to support PA for each person. JustWalk is based on Social Cognitive Theory (SCT) with N-of-1 adaptation driven by a mathematical dynamical model of SCT, which we have developed and validated. JustWalk can perform N-of-1 adaptation based on our innovative use of control engineering methods, which we call a control optimization trial (COT). We have a digital platform and empirical justification for our next step: to evaluate, in a randomized controlled trial (RCT), whether using a COT approach to continuously optimize a PA intervention to each individual is superior to an intervention that is identical but lacks the COT methods. Primary purpose: Evaluate differences in minutes/week of moderate-to-vigorous intensity PA (MVPA) among the COT- optimized vs. non-COT groups at 12 months. Hypotheses: We hypothesize significantly higher minutes/week of MVPA in the intervention arm (COT) relative to control (non-COT) as measured via ActiGraph (powered for effect size of ≥0.32). Methods: We will conduct this RCT with 386 adults aged 40+ who are inactive and overweight/obesity. This is a high-risk group who would benefit from a PA intervention for cancer prevention and who would benefit from an adaptive intervention because of the idiosyncratic and dynamic nature of PA that is pronounced within this group. Assessments will be conducted at baseline, 6, and 12-months using a hip-worn ActiGraph for assessing minutes/week of MVPA, as justified by guidelines. Implications: This research is highly significant because our intervention would be the first scalable PA intervention squarely grounded in

SCT with N-of-1 adaptation driven by a mathematical dynamical model version of SCT. Further, favorable results would justify use of our COT methods for other complex and highly idiosyncratic and dynamic behaviors such as weight management, smoking, or substance abuse. Finally, our work should improve understanding of engagement with digital health tools. This research is highly innovative as we would be the first to conduct a COT and to empirically evaluate its utility in an RCT.

PUBLIC HEALTH RELEVANCE: Physical activity can reduce risk for a variety of chronic diseases including several types of cancer but, unfortunately, many individuals try but fail to be active (e.g., think New Year's Resolutions). The purpose of this project is to evaluate if an intervention that uses methods from control systems engineering to adjust support to each person's changing needs will promote increased physical activity compared to another intervention that does not use these methods.

CRITIQUE 1

Significance: 2
Investigator(s): 1
Innovation: 2
Approach: 2
Environment: 1

Overall Impact: This study proposes as novel RCT to compare COT to non-COT across a 12 month intervention to examine the effects on physical activity. Addressing concerns related to physical activity engagement and sustainability are of public health importance and providing novel approaches such as COT that include a N-of-1 approach may provide an effective approach. This study is based on extensive prior work, that in many areas is novel, that has been conducted by the investigators. Given the complexity of the COT technology, this was a well-written proposal with clearly defined aims. There are appropriate measures of physical activity and other measures of interest; however, the need to include HbA1c is not well justified. Moreover, the continued focus on only including adults with overweight or obesity continues to be of minor concern. While there may also be addition concerns, these are deemed to be minor and likely addressable by the investigators. Thus, this is a novel application that has the potential for high impact related to physical activity participation, and the investigators have been response to the prior review. This heightens the enthusiasm for this application.

1. Significance:

Strengths

- This study is focused on enhancing engagement in physical activity, which has been shown to be an important lifestyle behavior across many health-related conditions.
- While some intervention approaches have shown promise, these have not addressed the individual-level variability in response. This study proposes to conduct a control optimization trial (COT), which incorporates an N-of-1 approach, in an attempt to further enhance the response and improve physical activity participation.
- The scientific rigor to justify the approach that is proposed is rated as high, and the investigators provide an extensive and well-balanced approach of the prior research and alternative study designs (e.g., SMART, etc.).

Weaknesses

- The intervention is sophisticated and requires ongoing engagement (e.g., daily) with the study participant. While this may be important within the context of a research study, in non-research participants there are many demands on time, and at times their focus may be on other important lifestyle factors that do not involve physical activity. Thus, long-term data on the feasibility of a COT approach will be of great importance.

2. Investigator(s):

Strengths

- The team led by Dr. Hekler is very strong. This team has engaged in many of the aspects that inform the study design and the technologies that are being proposed.
- This is a MPI application with Dr. Rivera, a control engineer, serving as a Co-PI.
- An appropriate MPI plan is provided. There is also a plan for how the team will deal with changes in investigators should that become necessary.
- Additional expertise is provided by other on the study team.

Weaknesses

- None noted by reviewer.

3. Innovation:

Strengths

- This study brings together a mHealth approach and components of behavioral theory that are focused on physical activity engagement.
- There is rigorous data driven optimization of the COT (N-of-1), which is a novel approach to physical activity interventions.

Weaknesses

- None noted by reviewer.

4. Approach:

Strengths

- This study is 12-months in duration to compare a COT to a non-COT approach for physical activity.
- Objective physical activity will be assessed using actigraphy at 0, 6, and 12 months, with additional ongoing physical activity data collection from a FitBit across the entire 12-month period.
- There are additional measures of weight, BMI, fitness, and other behavioral measures based primarily on social cognitive theory.
- The COT is focused on goal setting, feedback, and reinforcement/reward. Within the context of space limitations of this application, the COT is adequately described and is based on control systems engineering.
- The COT and non-COT platforms have been established by the investigators and is therefore ready for use in this study with potentially minor modifications as deemed necessary by the investigators based on their prior work.

- Measures of usability and acceptability are included.

Weaknesses

- The need for assessment of HbA1c is not justified. It is unclear why this measurement is included in this study.
- Given the importance of physical activity regardless of body fatness and body weight, the continued focused on only recruiting participants with overweight or obesity is not justified.
- Given the potential health concerns with individuals with higher levels of obesity, the investigators should consider ECG measures of heart rate and rhythm during the exercise test rather than the use of a Polar system to assess heart rate.
- Given that the study is not specifically focused on obesity or weight loss, it is unclear why individuals will be excluded if taking a stable dose of prescription medication that may alter body weight.
- Further justification that the remuneration provided at 0, 6, and 12 month will be sufficient to maximize retention should be provided given the participant burden based on the assessments that will be performed.

5. Environment:

Strengths

- The facilities and resources at UC San Diego, Arizona State University, and the University of Michigan are outstanding to perform the study as currently designed.

Weaknesses

- None noted by reviewer.

Study Timeline:

Strengths

- The study timeline appears to be appropriate given the scope of the work proposed.

Weaknesses

- None noted by reviewer.

Protections for Human Subjects:

Unacceptable Risks and/or Inadequate Protections

- It is recommended that the investigators consider using ECG during the exercise tests to ensure participant safety.

Data and Safety Monitoring Plan (Applicable for Clinical Trials Only):

Acceptable

- o A DSMP is provided and appears to be adequate.

Inclusion Plans:

- Sex/Gender: Distribution justified scientifically

- Race/Ethnicity: Distribution justified scientifically
- For NIH-Defined Phase III trials, Plans for valid design and analysis: Not applicable
- Inclusion/Exclusion Based on Age: Distribution justified scientifically
- Both men and women, from a diverse race/ethnic groups, and across a broad age range are eligible to participate in this study.

Vertebrate Animals:

Not Applicable (No Vertebrate Animals)

Biohazards:

Not Applicable (No Biohazards)

Revision:

- This study was very responsive to a prior review. The investigators considered the prior comments and where appropriate adjusted their approach, which has strengthened this application. They have also provided adequate responses to concerns raised about the length of the study, make-up of the study team, age inclusion and many of the aspects of the prior review.

Resource Sharing Plans:

Acceptable

Budget and Period of Support:

Budget Modifications Recommended (in amount/time)

Recommended budget modifications or possible overlap identified:

- Inclusion of the HbA1c measures are not justified.

CRITIQUE 2

Significance: 2
Investigator(s): 1
Innovation: 2
Approach: 2
Environment: 1

Overall Impact: This application uses an adaptive PA walking intervention incorporating a control optimization design (COT) to increasing MVPA among a sample of inactive, overweight or obese adults. The project addresses a significant, novel scientific premise and displays outstanding scientific rigor. The proposed COT that could have a meaningful impact in optimizing PA promotion efforts in this vulnerable population. The proposed COT intervention is a promising, novel approach that has considerable reach and scalability potential. If the aims are achieved, the project has potential for a

meaningful impact. Overall, the investigators were quite responsive to the concerns raised in the initial critique and this has strengthened the application considerably. The application is well-written, there is merit and innovation to research objectives, and the approach is well-designed and appropriate to achieve the project aims. A well-qualified investigative team with complementary expertise has been assembled and is led by a strong principal investigator with considerable experience in mHealth PA interventions. The environment is outstanding and clearly provides the resources necessary to successfully complete the project. Overall, the application has several notable strengths, and possesses potential for meaningful public health impact. Select minor, addressable concerns remain including: (a) relatively limited focus is placed on barrier problem-solving and harnessing social support; and (b) Whereas removing the 18 mo follow-up is responsive to the initial critique and reasonable given the present state of research knowledge, it also raises some ancillary concerns regarding what can be concluded about long-term maintenance of MVPA. Nevertheless, these are minor addressable issues. The proposed project is a well-written, ambitious application with a number of significant and novel features and potential for meaningful impact.

1. Significance:

Strengths

- The investigators were quite responsive to the concerns raised in the initial critique and this has strengthened the application considerably. The revision to focus on MVPA as the primary outcome is far more compelling.
- The application is well-written, there is merit and innovation to research objectives
- The study rationale and scientific premise are clearly articulated and well justified.
- A well-qualified investigative team with complementary expertise, led by a strong principal investigator has been assembled. The project builds logically upon the team’s prior mHealth and PA promotion work and heightens potential for project success
- The study targets a vulnerable, priority population of overweight adults at risk for chronic disease who could benefit considerably from an mhealth PA intervention.
- Implementing the COT framework to promote optimization of the mhealth, technology-supported PA intervention is significant, quite innovative, and well-justified.
- The proposed COT intervention is a promising, novel approach that has considerable reach and scalability potential.
- The proposed approaches are generally well-designed and appropriate to evaluate the primary project objectives.

Weaknesses

- Only minor weakness are evident. Although the additional description of the BCTs is quite instructive, it appears as though a relatively limited focus is placed on barrier problem-solving and harnessing social support. More explicitly addressing these strategies in the action planning aspects of the intervention and how the COT can be adapted to address these issues would be informative.
- Whereas removing the 18 mo follow-up is responsive to the initial critique and reasonable given the present state of research knowledge, it also raises some ancillary concerns regarding what can be concluded about long-term maintenance of MVPA.

2. Investigator(s):

Strengths

- A well-qualified investigative team with complementary expertise has been assembled and is led by a strong PI with considerable experience in mHealth PA interventions.
- The project builds logically upon the team's strong preliminary work and heightens potential for project success

Weaknesses

- None noted by reviewer.

3. Innovation:

Strengths

- The mhealth, technology-supported COT approach is novel and has potential to meaningfully expanding access, reach, and scalability in PA promotion.
- The study targets a vulnerable, priority population of overweight adults at risk for chronic disease who could benefit considerably from an mhealth PA intervention.
- Implementing the COT framework to promote optimization of the mhealth, technology-supported PA intervention is significant, quite innovative, and well-justified.
- The proposed approaches are generally well-designed and appropriate to evaluate the primary project objectives.

Weaknesses

- None noted by reviewer.

4. Approach:

Strengths

- There is strong merit and innovation to research objectives.
- Implementing the COT approach to optimize of the mhealth, technology-supported PA intervention is a significant and appropriate to achieve the project aims.
- The proposed approach is generally well-designed and characterized by strong scientific rigor.
- Use of established protocols that have been implemented in the teams' prior work and together with advanced, refined procedures heightens the likelihood for project success.
- A well-qualified investigative team with complementary expertise has been assembled.
- The project builds logically upon the team's strong preliminary work and heightens potential for project success.
- A clear, well-articulated theoretical foundation for the PA intervention is provided.

Weaknesses

- Only minor weaknesses are evident. Although the additional description of the BCTs is quite instructive, it appears as though a relatively limited focus is placed on barrier problem-solving and harnessing social support. More explicitly addressing these strategies in the action planning aspects of the intervention and how the COT can be adapted to address these issues would be informative.

- Whereas removing the 18 mo follow-up is responsive to the initial critique and reasonable given the present state of research knowledge, it also raises some ancillary concerns regarding what can be concluded about long-term maintenance of MVPA.

5. Environment:

Strengths

- The environment is outstanding. It provides appropriate access to the target sample and more than adequate resources and support necessary to successfully complete the proposed project.

Weaknesses

- None noted by reviewer.

Study Timeline:

Strengths

- Well organized and appropriate with adequate time proposed for relevant study activities.

Weaknesses

- None noted by reviewer.

Protections for Human Subjects:

Acceptable Risks and/or Adequate Protections

Data and Safety Monitoring Plan (Applicable for Clinical Trials Only):

- Acceptable

Inclusion Plans:

- Sex/Gender: Distribution justified scientifically
- Race/Ethnicity: Distribution justified scientifically
- For NIH-Defined Phase III trials, Plans for valid design and analysis: Not applicable
- Inclusion/Exclusion Based on Age: Distribution justified scientifically

Vertebrate Animals:

Not Applicable (No Vertebrate Animals)

Biohazards:

Not Applicable (No Biohazards)

Budget and Period of Support:

Recommend as Requested

CRITIQUE 3

Significance: 2
Investigator(s): 1
Innovation: 1
Approach: 2
Environment: 1

Overall Impact: This is a resubmission of an R01 application to evaluate a modular adaptive mobile health intervention to promote and support increased physical activity (PA) among mid-life adults (aged 40+) who are inactive and overweight or obese. Using a control optimization trial (COT) approach, the intervention takes into account various individual and contextual factors in order to continuously adjust the intervention program messages, goal setting, and rewards. Using a randomized controlled study design, the intervention effects on minutes/week of moderate to vigorous physical activity (MVPA), steps/day, psychosocial constructs, and biomarkers of health and cardiorespiratory fitness will be assessed. Key strengths of this application include the innovation of using an adaptive design that has potential to be more engaging and motivational, an experienced team of investigators who have successfully collaborated on prior related projects, and the randomized study design with a control group. In particular, pilot data demonstrate that this intervention is acceptable and shows promising effects for increasing PA. The proposed study design and methodology contribute to high scientific rigor of this application. However, the rationale for the selection of several outcome measures was unclear, as these are not well-integrated with the scientific premise of the study; but these were considered to be minor weaknesses in an otherwise strong application.

1. Significance:

Strengths

- Scalable interventions to promote and maintain physical activity (PA) have significant potential to broadly improve public health.
- The application clearly describes the rigor of the prior research and addresses limitations in the literature by incorporating an N-of-1 approach, which may be more effective than prior interventions because it can take into account how various factors impact each individual.

Weaknesses

- As the proposal is generally focused on the link between PA and cancer risk, the rationale for including some of the secondary outcomes (HbA1c, V02 submax) -- as opposed to other markers that have a more direct association with cancer risk, such as inflammation -- is weak and not well-justified.

2. Investigator(s):

Strengths

- The PI is a clinical psychologist with extensive training and experience in the development of physical activity interventions. He also has expertise in digital health technologies (mHealth) and has prior experience using control systems engineering methods.
- The MPI (Rivera) is a chemical engineer with expertise in the field of control systems engineering. He has worked with the PI (Hekler) on related studies. They have demonstrated a well-established collaborative relationship.

- Other team members include co-investigators with expertise in biostatistics, health technologies, epidemiology and measurement, research ethics, and family medicine.

Weaknesses

- No major weaknesses. Prior concerns about the composition of the study team have been adequately addressed.

3. Innovation:

Strengths

- Evaluation of a COT-optimized intervention (using daily N-of-1 data driven adjustments) is innovative.
- Using a thinning reinforcement schedule (during the maintenance phase) is novel.

Weaknesses

- None noted.

4. Approach:

Strengths

- The modular adaptive mHealth intervention (JustWalk) is based on Social Cognitive Theory and incorporates daily N-of-1 adjustments to personally support and promote behavior change.
- The intervention can take into account other factors (weather, busyness) that impact PA behaviors.
- Pilot data demonstrates high acceptability of the intervention and preliminary efficacy.
- Objective measures of MVPA are a strength as these are linked to chronic disease risk, not subject to participant recall issues, and have been widely used in other trials.
- Randomized controlled trial study design will allow direct comparison of the COT-optimized intervention, which enhances the scientific rigor of the proposed project.

Weaknesses

- Use of single-item questions to assess SCT constructs is less than ideal. (Minor)
- Large number of patient-reported outcomes to be assessed, but not well justified or integrated with scientific rationale. (Minor)

5. Environment:

Strengths

- The environment at UCSD is excellent and can support the proposed work.
- Collaborating institutions have the resources needed to carry out their specific activities.
- Letter of support from Matrix Research, Inc. supports the feasibility of achieving the proposed accrual goal.

Weaknesses

- No major concerns.

Study Timeline:

Strengths

- The study timeline appears reasonable for the proposed activities.

Weaknesses

- No major weaknesses.

Protections for Human Subjects:

Acceptable Risks and/or Adequate Protections

- Potential risks are described and adequately addressed.

Data and Safety Monitoring Plan (Applicable for Clinical Trials Only):

Acceptable

- The DSM Plan is appropriate for the relatively low-risk trial being proposed.

Inclusion Plans:

- Sex/Gender: Distribution justified scientifically
- Race/Ethnicity: Distribution justified scientifically
- For NIH-Defined Phase III trials, Plans for valid design and analysis: Not applicable
- Inclusion/Exclusion Based on Age: Distribution justified scientifically
- Eligible participants include mid-life adults, both men and women, who are PA-deficient. Race/ethnic representation will reflect national statistics and include over 50% racial/ethnic minorities.

Vertebrate Animals:

Not Applicable (No Vertebrate Animals)

Biohazards:

Not Applicable (No Biohazards)

Resubmission:

- The revised application has been responsive to initial concerns including updating the primary outcome measure, providing greater detail about the intervention and the recruitment firm, and adjusting personnel on the team. These changes have strengthened the overall application.

Resource Sharing Plans:

Acceptable

Budget and Period of Support:

Recommend as Requested

THE FOLLOWING SECTIONS WERE PREPARED BY THE SCIENTIFIC REVIEW OFFICER TO SUMMARIZE THE OUTCOME OF DISCUSSIONS OF THE REVIEW COMMITTEE, OR REVIEWERS' WRITTEN CRITIQUES, ON THE FOLLOWING ISSUES:

PROTECTION OF HUMAN SUBJECTS: UNACCEPTABLE. The investigators may want to consider using ECG during the exercise tests to ensure participant safety.

INCLUSION OF WOMEN PLAN: ACCEPTABLE

INCLUSION OF MINORITIES PLAN: ACCEPTABLE

INCLUSION ACROSS THE LIFESPAN PLAN: ACCEPTABLE

COMMITTEE BUDGET RECOMMENDATIONS: The budget was recommended as requested.

Footnotes for 1 R01 CA244777-01A1; PI Name: Hekler, Eric B

NIH has modified its policy regarding the receipt of resubmissions (amended applications). See Guide Notice NOT-OD-14-074 at http://grants.nih.gov/grants/guide/notice-files/NOT-OD-14-074.html. The impact/priority score is calculated after discussion of an application by averaging the overall scores (1-9) given by all voting reviewers on the committee and multiplying by 10. The criterion scores are submitted prior to the meeting by the individual reviewers assigned to an application, and are not discussed specifically at the review meeting or calculated into the overall impact score. Some applications also receive a percentile ranking. For details on the review process, see http://grants.nih.gov/grants/peer_review_process.htm#scoring.

MEETING ROSTER

Psychosocial Risk and Disease Prevention Study Section
Risk, Prevention and Health Behavior Integrated Review Group
CENTER FOR SCIENTIFIC REVIEW
PRDP
01/27/2020 - 01/28/2020

Notice of NIH Policy to All Applicants: Meeting rosters are provided for information purposes only. Applicant investigators and institutional officials must not communicate directly with study section members about an application before or after the review. Failure to observe this policy will create a serious breach of integrity in the peer review process, and may lead to actions outlined in NOT-OD-14-073 at https://grants.nih.gov/grants/guide/notice-files/NOT-OD-14-073.html and NOT-OD-15-106 at https://grants.nih.gov/grants/guide/notice-files/NOT-OD-15-106.html, including removal of the application from immediate review.

CHAIRPERSON(S)

JAKICIC, JOHN M, PHD
PROFESSOR
DEPARTMENT OF HEALTH AND HUMAN DEVELOPMENT
UNIVERSITY OF PITTSBURGH
PITTSBURGH, PA 15261

MEMBERS

APPELHANS, BRADLEY M, PHD
ASSOCIATE PROFESSOR
DEPARTMENT OF PREVENTIVE MEDICINE
RUSH UNIVERSITY MEDICAL CENTER
CHICAGO, IL 60612

BEETS, MICHAEL W, PHD *
PROFESSOR
DEPARTMENT OF EXERCISE SCIENCE
ARNOLD SCHOOL OF PUBLIC HEALTH
UNIVERSITY OF SOUTH CAROLINA
COLUMBIA, SC 29208

BILLIMEK, JOHN, PHD *
ASSOCIATE PROFESSOR
DEPARTMENT OF FAMILY MEDICINE AND HEALTH POLICY
UNIVERSITY OF CALIFORNIA, IRVINE
IRVINE, CA 92697

BUMAN, MATTHEW P, PHD
ASSOCIATE PROFESSOR
COLLEGE OF HEALTH SOLUTIONS
ARIZONA STATE UNIVERSITY
PHOENIX, AZ 85004

CHEN, EUNICE YU, PHD *
ASSOCIATE PROFESSOR
DEPARTMENT OF PSYCHOLOGY
COLLEGE OF LIBERAL ARTS
TEMPLE UNIVERSITY
PHILADELPHIA, PA 19122

DUTTON, GARETH R, PHD
PROFESSOR
DIVISION OF PREVENTIVE MEDICINE
DEPARTMENT OF MEDICINE
UNIVERSITY OF ALABAMA AT BIRMINGHAM
BIRMINGHAM, AL 35205

FANG, CAROLYN Y, PHD *
PROFESSOR AND CO-LEADER
CANCER PREVENTION AND CONTROL PROGRAM
FOX CHASE CANCER CENTER
PHILADELPHIA, PA 19111

FITZPATRICK, STEPHANIE LENAY, PHD
INVESTIGATOR
CENTER FOR HEALTH RESEARCH
KAISER FOUNDATION RESEARCH INSTITUTE
PORTLAND, OR 97227

FOCHT, BRIAN CARL, PHD
PROFESSOR
DEPARTMENT OF HUMAN SCIENCES
COLLEGE OF EDUCATION AND HUMAN ECOLOGY
THE OHIO STATE UNIVERSITY
COLUMBUS, OH 43210

FRANCIS, LORI ANNE, PHD
ASSOCIATE PROFESSOR
DEPARTMENT OF BIOBEHAVIORAL HEALTH
PENNSYLVANIA STATE UNIVERSITY
UNIVERSITY PARK, PA 16802

FUEMMELER, BERNARD F, PHD
PROFESSOR
DEPARTMENT OF HEALTH BEHAVIOR AND POLICY
MASSEY CANCER CENTER
VIRGINIA COMMONWEALTH UNIVERSITY
RICHMOND, VA 23298

HART, CHANTELLE NOBILE, PHD
ASSOCIATE PROFESSOR
DEPARTMENT OF SOCIAL AND BEHAVIORAL SCIENCES
CENTER FOR OBESITY RESEARCH AND EDUCATION
TEMPLE UNIVERSITY
PHILADELPHIA, PA 19140

HARVEY, JEAN R, PHD
PROFESSOR AND CHAIR
DEPARTMENT OF NUTRITION AND FOOD SCIENCES
UNIVERSITY OF VERMONT
BURLINGTON, VT 05405

LEWIS, BETH A, PHD
PROFESSOR AND DIRECTOR
SCHOOL OF KINESIOLOGY
UNIVERSITY OF MINNESOTA
MINNEAPOLIS, MN 55455

LEWIS, MEGAN A, PHD *
PROGRAM DIRECTOR
CENTER FOR COMMUNICATION SCIENCE
RTI INTERNATIONAL
RESEARCH TRIANGLE PARK, NC 27709

LUMENG, JULIE C, MD
PROFESSOR
DEPARTMENT OF PEDIATRICS
UNIVERSITY OF MICHIGAN, ANN ARBOR
ANN ARBOR, MI 48109

MCVAY, MEGAN APPERSON, PHD *
ASSISTANT PROFESSOR
DEPARTMENT OF HEALTH EDUCATION AND BEHAVIOR
UNIVERSITY OF FLORIDA
GAINESVILLE, FL 32611

MENDOZA, JASON A, MD
FULL MEMBER AND ASSOCIATE PROGRAM HEAD
CANCER PREVENTION PROGRAM
PUBLIC HEALTH SCIENCE DIVISION
FRED HUTCHINSON CANCER RESEARCH CENTER
SEATTLE, WA 98109

NEELON, SARA ELIZABETH, PHD
ASSOCIATE PROFESSOR
DEPARTMENT OF HEALTH
BEHAVIOR AND SOCIETY
JOHNS HOPKINS SCHOOL OF PUBLIC HEALTH
BALTIMORE, MD 21205

NELSON, TIMOTHY DAVID, PHD *
PROFESSOR
DEPARTMENT OF PSYCHOLOGY
UNIVERSITY OF NEBRASKA-LINCOLN
LINCOLN, NE 68588

NEWTON, ROBERT LEE JR, PHD
ASSOCIATE PROFESSOR
POPULATION AND PUBLIC HEALTH
PENNINGTON BIOMEDICAL RESEARCH CENTER
BATON ROUGE, LA 70808

O'CONNOR, TERESIA MARGARETA, MD, MPH *
ASSOCIATE PROFESSOR
DEPARTMENT OF PEDIATRICS
BAYLOR COLLEGE OF MEDICINE
HOUSTON, TX 77030

RAYNOR, HOLLIE A, PHD
PROFESSOR
DEPARTMENT OF NUTRITION
UNIVERSITY OF TENNESSEE
KNOXVILLE, TN 37996

RHEE, KYUNG E, MD, MA
ASSOCIATE PROFESSOR
DEPARTMENT OF PEDIATRICS
UNIVERSITY OF CALIFORNIA, SAN DIEGO
LA JOLLA, CA 92093

SAELENS, BRIAN E, PHD *
PROFESSOR
CENTER FOR CHILD HEALTH, BEHAVIOR
AND DEVELOPMENT
SEATTLE CHILDREN'S HOSPITAL RESEARCH INSTITUTE
UNIVERSITY OF WASHINGTON
SEATTLE, WA 98101

SALVY, SARAH-JEANNE, PHD *
ASSOCIATE PROFESSOR
DEPTARTMENT OF MEDICINE
SAMUEL OSCHIN COMPREHENSIVE CANCER INSTITUTE
CEDARS SINAI MEDICAL CENTER
LOS ANGELES, CA 90048

SCHNEIDER, MARGARET L, PHD
PROFESSOR
DEPARTMENTS OF POPULATION HEALTH AND
DISEASE PREVENTION
INSTITUTE FOR CLINICAL AND TRANSLATIONAL SCIENCE
UNIVERSITY OF CALIFORNIA, IRVINE
IRVINE, CA 92617

SHAY, LAURA AUBREE, PHD *
ASSISTANT PROFESSOR
DEPARTMENT OF HEALTH PROMOTION
AND BEHAVIORAL SCIENCE
UTHEALTH SCHOOL OF PUBLIC HEALTH IN SAN ANTONIO
SAN ANTONIO, TX 78229

STOLLEY, MELINDA R, PHD
PROFESSOR
DEPARTMENT OF MEDICINE
MEDICAL COLLEGE OF WISCONSIN
MILWAUKEE, WI 53226

TEMPLE, JENNIFER L, PHD
ASSOCIATE PROFESSOR
DEPARTMENTS OF EXERCISE AND NUTRITION SCIENCES
AND COMMUNITY HEALTH AND HEALTH BEHAVIOR
SCHOOL OF PUBLIC HEALTH AND HEALTH PROFESSIONS
UNIVERSITY AT BUFFALO
BUFFALO, NY 14214

WEISSMAN, RUTH STRIEGEL, DIPL, PHD *
PROFESSOR
DEPARTMENT OF PSYCHOLOGY
WESLEYAN UNIVERSITY
MIDDLETOWN, CT 06459

WILLIAMS, DAVID M, PHD
ASSOCIATE PROFESSOR
DEPARTMENT OF BEHAVIORAL AND SOCIAL SCIENCES
CENTER FOR HEALTH EQUITY RESEARCH
BROWN UNIVERSITY SCHOOL OF PUBLIC HEALTH
PROVIDENCE, RI 02912

SCIENTIFIC REVIEW OFFICER

FITZSIMMONS, STACEY, PHD
SCIENTIFIC REVIEW OFFICER
CENTER FOR SCIENTIFIC REVIEW
NATIONAL INSTITUTES OF HEALTH
BETHESDA, MD 20892

EXTRAMURAL SUPPORT ASSISTANT

FAYEMIWO, TOLU, MS
EXTRAMURAL SUPPORT ASSISTANT
CENTER FOR SCIENTIFIC REVIIEW
NATIONAL INSTITUTES OF HEALTH
BETHESDA, MD 20892

* Temporary Member. For grant applications, temporary members may participate in the entire meeting or may review only selected applications as needed.

Consultants are required to absent themselves from the room during the review of any application if their presence would constitute or appear to constitute a conflict of interest.
